# Revisiting climate impacts of an AMOC slowdown: dependence on freshwater locations in the North Atlantic

**DOI:** 10.1126/sciadv.adr3243

**Published:** 2024-11-20

**Authors:** Qiyun Ma, Xiaoxu Shi, Patrick Scholz, Dmitry Sidorenko, Gerrit Lohmann, Monica Ionita

**Affiliations:** ^1^ Alfred Wegner Institute Helmholtz Center for Polar and Marine Research, Bremerhaven, Germany.; ^2^ Southern Marine Science and Engineering Guangdong Laboratory, Zhuhai, China.; ^3^ Faculty of Physics & MARUM, University of Bremen, Bremen, Germany.; ^4^ Faculty of Forestry, “Stefan cel Mare” University of Suceava, Suceava, Romania.

## Abstract

The key locations of freshwater input driving Atlantic Meridional Overturning Circulation (AMOC) slowdown and their climate responses remain inconclusive. Using a state-of-the-art global climate model, we conduct freshwater hosing experiments to reexamine AMOC sensitivity and its climate impacts. The Irminger basin emerges as the most effective region for additional freshwater fluxes, causing the greatest AMOC weakening. While global temperature and precipitation responses are relatively homogeneous, subcontinental responses—especially in the northern mid-latitudes—are heterogeneous. At high latitudes, sea ice responses to freshwater fluxes and associated ice-albedo feedbacks determine temperature changes. In tropical and extratropical regions, temperature dynamics are shaped by atmospheric circulation and oceanic heat transport. Precipitation shows seasonal and regional variability due to altered surface turbulent heat flux and the southward movement of the Intertropical Convergence Zone (ITCZ). The widespread heterogeneity in climate extremes underscores the need to monitor freshwater release regions linked to AMOC slowdown. These findings hold vital implications for understanding paleoclimate and future AMOC impacts.

## Teaser

Freshwater input location strongly influences AMOC slowdown, with the Irminger basin having the greatest effect.

## INTRODUCTION

The change in the Atlantic Meridional Overturning Circulation (AMOC) stands as a pivotal tipping element of Earth system, substantially influencing global climate by redistributing vast amounts of energy (*1*–*5*). The shutdown of this circulation has been associated with past abrupt climate changes [e.g., as evident in paleoclimate records (*6*, *7*), such as playing crucial role in driving glacial-interglacial transitions (*8*–*10*)]. Continuous monitoring indicates a current decline in AMOC strength in the North Atlantic, although the interpretation of this trend is subject to debate due to natural climate variability (*11*–*13*). Nonetheless, some contemporary proxies support the argument of the AMOC weakening, suggesting that its current intensity is the lowest in the past few hundred years (*12*, *14*). Amid anthropogenic warming, advanced climate models project a continued AMOC weakening throughout the 21st century (*15*), yet an abrupt collapse within this timeframe seems unlikely, as per the findings of the Sixth Assessment Report of the Intergovernmental Panel on Climate Change (IPCC AR6) (*16*). Nevertheless, the potential for an AMOC collapse remains beyond this century if greenhouse gas emissions continue to rise (*17*).

Climate impacts induced by an AMOC slowdown have been widely explored through numerical experiments (*2*, *3*, *17*–*19*). These experiments, known as water-hosing experiments, involve the introduction of additional freshwater fluxes uniformly distributed over deep water formation regions to artificially weaken the AMOC in climate models (*20*–*23*). The subpolar North Atlantic is a common region for conducting water-hosing experiments due to its role as a deep water formation region for the North Atlantic Deep Water (NADW), a crucial component of the AMOC. Results from water-hosing experiments over the North Atlantic consistently indicate that a shutdown or weakening of the AMOC leads to large-scale climate impacts, including a cooling across the Northern Hemisphere (NH), an expansion of Arctic sea ice, and a southward shift of the Intertropical Convergence Zone (ITCZ). However, the homogeneity and heterogeneity of regional climate responses to an AMOC slowdown remain unclear, yet they are crucial for a better understanding of paleoclimatic records and the assessment of regional societal impacts.

Emerging research on the potential attribution of AMOC slowdown provides a scientific basis for conducting numerical water-hosing experiments. The location and transport patterns of input freshwater fluxes may lead to different climatic responses (*21*, *22*, *24*, *25*). Several factors have been proposed as potential freshwater sources for the future decline of the AMOC, including the reduction of Arctic sea ice (*26*) and melting land ice sheets around Greenland (*27*). The concurrent decline of Arctic sea ice and AMOC variations may conceal a two-way interaction between these processes, as suggested in (*28*). Arctic sea ice loss can weaken the AMOC after a multi-decadal delay, primarily through the downstream propagation of positive buoyancy anomalies to the subpolar North Atlantic (*29*). Conversely, an AMOC weakening can expand Arctic sea ice cover within several years by reducing northward heat transport. This interaction could operate independently of anthropogenic forcing, as an AMOC slowdown can mitigate Arctic sea ice loss and delay the onset of summer ice-free conditions in a warming climate (*3*).

However, the role of land ice melting is still uncertain. Many climate models predict a weakened AMOC even without fully considering freshwater fluxes from melting land ice (*2*, *30*). From the perspective of paleoclimate records, the influx of freshwater into the North Atlantic is believed to be a key driver of the most abrupt historical changes in the AMOC (*6*, *25*, *31*). However, identifying the crucial regions where an increase in freshwater fluxes leads to the substantial decline in the AMOC is still a topic of ongoing discussion and lacks consensus based on both observations and climate models (*24*, *31*–*34*). In addition, current climate models from the Coupled Model Intercomparison Project Phase 6 (CMIP6) show different abilities to capture and project AMOC strength, leading to considerable uncertainties between the models. All in all, identifying key regions that drive AMOC variability could enhance climate models by improving regional resolution and accounting for the associated physical and dynamical processes.

In this study, we use a state-of-the-art global climate model, the Alfred Wegener Institute Climate Model (AWI-CM3) (*35*), to reexamine the sensitivity of the AMOC to various water-hosing regions in the North Atlantic. This investigation is motivated by recent observations emphasizing the importance of the Irminger Sea in influencing AMOC dynamics (*34*, *36*). We explore both the homogeneous and heterogeneous climate impacts associated with an AMOC slowdown across different water-hosing regions.

## RESULTS

### AMOC in control simulations

**Fig. 1.**
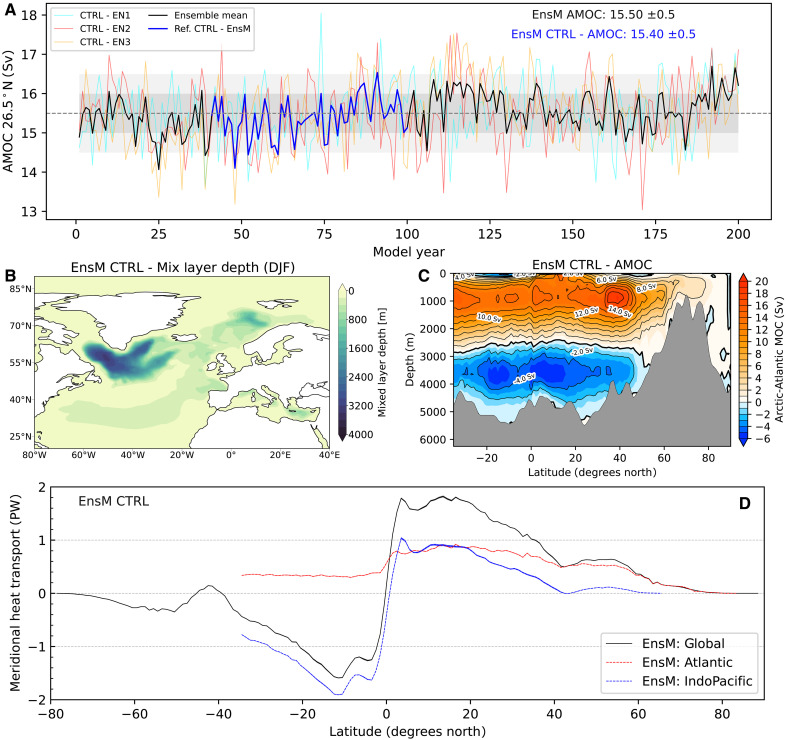
Characteristics of AMOC and poleward heat transport in control simulations. (**A**) Annual-mean time series of AMOC strength (maximum in depth at 26.5°N) across all control experiment ensemble members, alongside the ensemble mean. The blue line represents the reference period, covering model years 40 to 99 (60 years). The gray dashed line denotes the ensemble-time mean of the simulated AMOC strength over the 200 years, with shading indicating SDs (darker for ±1σ, lighter for ±2σ). Ensemble mean of (**B**) the annual mean AMOC stream functions (Sverdrups) and (**C**) mixed layer depth (m) in boreal winter [December, January, and February (DJF)] during the reference period in the control simulation. (**D**) Meridional heat transport during the reference period; shading indicates the ensemble spread as measured by the ±1σ.

Figure 1A presents the simulated AMOC in the pre-industrial control (PI-CTR) experiments for each ensemble member and the ensemble mean. The AMOC strength is measured by the annual maximum of the overturning stream function below 500 m in the North Atlantic at 26.5°N. The ensemble time-mean AMOC strength and one SD (σ) is approximately 15.5 ± 0.5 Sverdrups (Sv) throughout the 200 model years. Comparing the pre-industrial simulations with other climate models, our simulated AMOC strength aligns with the range reported across various climate models (*23*, *37*, *38*), yet this value is slightly below what recent observational studies suggest (*39*).

Notably, the ensemble mean AMOC strength shows a minor upward trend, increasing by 0.2 Sv per century (*P* < 0.01), likely due to temperature drift in the ocean model (*35*). Interannual fluctuations among the three ensemble members mostly fall within 2σ of the long-term ensemble time mean. For our analysis, we set the reference period from year 40 to 99, where the AMOC strength averages around 15.4 ± 0.5 Sv. There is also a modest increasing trend approximately 0.1 Sv per century (*P* < 0.01). Testing different reference periods yielded consistent outcomes, confirming the robustness of our choice. Therefore, subsequent discussions of control simulations and differences are based on the defined reference period.

Figure 1B presents the AMOC stream function within the Atlantic/Arctic sector over the reference period, displaying a realistic modern-day overturning circulation (*2*). This includes northward transport in the upper 1000 m, on average, with water sinking in the high-latitude North Atlantic before returning southward in a flow between 1000 and 3000 m (*23*). The winter season [December, January, and February (DJF)] mixed layer depth, depicted in Fig. 1C, identifies critical regions of deep water formation in the North Atlantic (*40*). These areas are essential for the formation of the NADW, contributing to the returning southwards flow of the AMOC. AWI-CM3 simulations pinpoint major NADW formation sites including the Labrador Sea, the Irminger Sea, southern Greenland, and the Greenland-Iceland-Norwegian seas, with the deepest mixed layer depths observed in the Labrador Sea (reaching approximately 3200 m). These depths present a bias from observational data, as highlighted in (*41*), although biases in the Nordic Seas and Irminger Sea are notably less pronounced.

We further explore oceanic meridional heat transport (MHT), as illustrated in Fig. 1D. Oceanic heat primarily dominates in the deep tropics and is transported toward higher latitudes, a well-documented characteristic shown in both observational data and climate model analyses (*42*, *43*). Further decomposing the MHT across various basins reveals that, in deep tropics, heat transports from the Indo-Pacific Ocean generally dominates in both hemispheres. Here, the Indo-Pacific’s contribution is more substantial in the Southern Hemisphere [SH; 1.8 petawatt (PW)] compared to the NH (0.9 PW). The MHT within the Atlantic demonstrates overall northward heat transport across the two hemispheres largely facilitated by the AMOC, aligning with its circulation patterns depicted in Fig. 1B. While the Atlantic substantially affects global MHT in the tropics, it also plays a pivotal role in MHT from mid-latitudes to high latitudes (∼75°N) within the NH. We estimate the Atlantic heat transport at 26.5°N to be around 0.81 ± 0.005 PW, falling short of the observed 1.33 ± 0.4 PW (*44*). Despite the challenges posed by global warming and the tendency for a low bias in AMOC simulations, our control simulation provides a credible estimation of the oceanic MHT associated with AMOC. Furthermore, our findings regarding oceanic MHT at both global and basin-specific scales display consistency across different ensemble members.

### Responses of AMOC in perturbed simulations

**Fig. 2.**
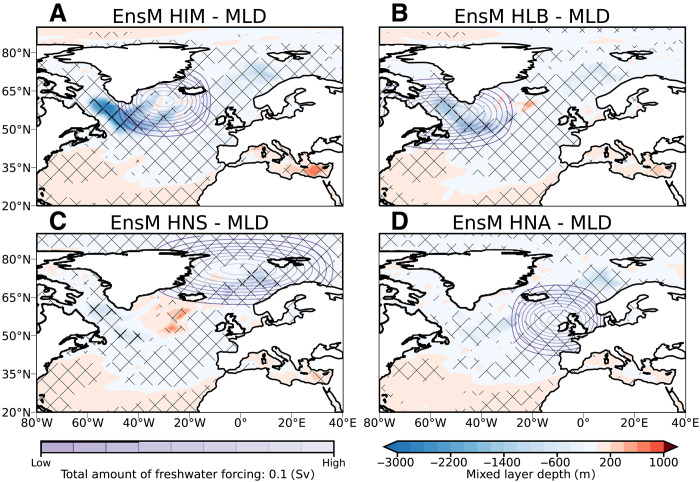
Four regions for water-hosing experiments and changes in wintertime mixed layer depth. The specified regions are delineated as contours and detailed in table S1: (**A**) Irminger basin (HIM), (**B**) Labrador Sea (HLB), (**C**) Nordic Seas (HNS), and (**D**) North-East Atlantic (HNA). Colored shading illustrates the changes in the wintertime mixed layer depths, indicating as the ensemble mean of the differences observed between the strongest decline period of AMOC and the control experiments. Cross-hatched areas indicate that the regional changes reach statistical significance at the 99% confidence level using a two-sided Student’s *t* test.

In response to freshwater perturbations across four designated input regions (Fig. 2 and Materials and Methods), we note a consistent pattern of initial AMOC strength reduction followed by a recovery phase in all experimental setups (text S1 and figs. S1 and S2). During the period influenced by freshwater, the AMOC strength diminishes to varying degrees, indicating that changes in AMOC variations depend on the freshwater input regions. It is essential to note that these responses are transient and have not reached a state of equilibrium, as expected due to the slow adjustment of the deep ocean (*45*). The recovery of AMOC strength, occurring about 50 years after ceasing freshwater injections, corresponds closely with the length of the perturbation period (fig. S1). The recovered AMOC (model year 100 to 200) can either reach the controlled strength or slightly surpass it, possibly due to the overshoot effect (text S1 and fig. S2). In this study, we mainly focus on the transient response of the weakening and recovering AMOC with different sensitive freshwater-perturbed regions during the initial 100 model years.

**Fig. 3.**
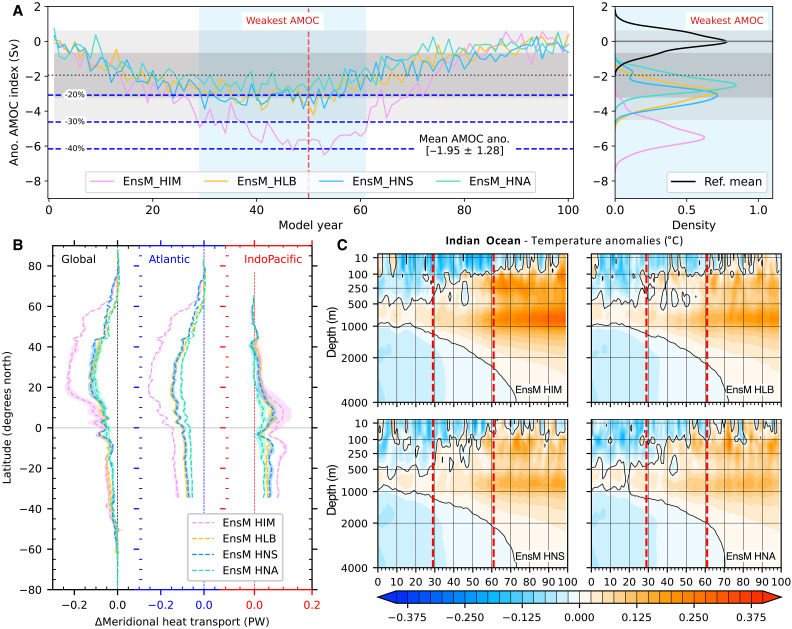
Variations in AMOC strength and poleward heat transport across water-hosing experiments. (**A**) Left panel shows the ensemble mean of AMOC intensity anomalies relative to the reference period. The black dotted line denotes the averaged of all the anomalies over the first 100 model years across all the water-hosing experiments, with gray shadings indicating the SD (darker for ±1σ, lighter for ±2σ). Blue horizontal lines mark the levels of AMOC weakening relative to the reference period. Light blue shading defines the strongest decline period, during which AMOC strength reduces by up to 2σ (approximately 30%) in HIM experiment. The right panel presents probability density functions of AMOC strength during the strongest decline period among different water-hosing experiments. (**B**) Anomalies in the meridional ocean heat transport averaging the strongest decline period of AMOC. (**C**) Hovmöller diagram of the annual mean temperature difference averaged in 0 to 4000-m ocean depth in the Indian Ocean basin relative to the reference period. The vertical red dashed lines indicate the section corresponding to the strongest decline period of AMOC.

Analysis of the anomalies in AMOC strength across different experiments reveal a reduction of 20 to 40% at the end of the freshwater perturbations compared to the control simulations (Fig. 3A). The largest decline in AMOC appears in the Hosing-Irminger (HIM) experiment, where the Irminger basin is hosed, while the minimal response is found in the Hosing-NorthEast-Atlantic (HNA), where freshwater is injected over the North-East Atlantic. The weakening of AMOC strength appears to occur at a similar pace in the Hosing-Labrador (HLB) and Hosing-Nordic (HNS), where freshwater fluxes are perturbed over the Labrador Sea and Nordic Seas, respectively. In agreement with previous research (*24*, *31*), our results demonstrate that changes in AMOC intensity are generally larger when freshwater is injected directly over the deep water formation regions (Fig. 1C). In addition, our findings contribute fresh insights, corroborating observational data that the Irminger basin exhibits a heightened sensitivity to fluctuations in AMOC strength (*34*, *36*). Observations in (*36*) noted that colder waters from the Labrador Sea, advected eastward by the subpolar gyre circulation closely controlled by the North Atlantic Oscillation, play a crucial role in driving the overturning dynamics within the Irminger basin.

To further investigate the climate response to a weakened AMOC, we define a period with the strongest decline in AMOC strength. This involves averaging AMOC strength anomalies across all water-hosing experiments over the initial 100 model years (Fig. 3A), resulting in an average anomaly of approximately −1.95 ± 1.28 Sv. We define the “strongest decline period” as the span in which the reduction of AMOC intensity falls below −2 σ, observed in experiment where the AMOC shows maximum responsiveness to freshwater perturbation. This critical period is determined to be from the years 29 to 61 (indicated by light blue shading in Fig. 3A). We note that the AMOC recovery phase begins after a lag of approximately 5 years following the cessation of freshwater hosing (year 50), which may be linked to the delayed responses between subpolar and subtropical AMOC variations via interior pathways and coastal Kelvin waves (*46*). Our subsequent analyses primarily focus on comparing climate responses during this period of the strongest AMOC weakening with the reference period across all water-hosing experiments.

During the period of the strongest AMOC decline, the difference in AMOC strength between the HIM and others is statistically significant (right panel in Fig. 3A; *P* < 0.01). While the decline in AMOC strength between the HLB and HNS is not statistically significant, the HLB tends to experience more years of extreme AMOC weakening, and the HNS shows a slightly greater overall decline, as indicated by their average values. The relatively smaller AMOC decline in HNA is also significantly different from other experiments (*P* < 0.01). All perturbed experiments result in a significant (*P* < 0.01) reduction in AMOC strength compared to the reference control experiments. In examining the structure of the AMOC cells, all experiments demonstrate a general weakening of the entire AMOC cell and a slight intensification of the bottom reversed cell (fig. S3). A notable observation is the shallowing of the upper AMOC cell, particularly in HIM, where the maximum stream function reduces to approximately 12 to 14 Sv at around 40°N (fig. S3A), marking a decrease of about 4 to 5 Sv.

### Reduced the North Atlantic deep water formation

The weakening of the upper AMOC cell is further evidenced by changes in wintertime mixed layer depth (Fig. 2). The largest decrease in the mixed layer depth is most clear for HIM over the Labrador Sea (Fig. 2A), where the climatological maximum mixed layer depth is located (Fig. 1C). This reduction leads to shallower deep water formation, correlating with the weakening of the AMOC. Echoing a similar model configuration, Sidorenko *et al.* (*47*) highlighted that deep convection in the North Atlantic predominantly occurs in the eastern sectors of the subpolar gyre or further north. This accounts for the greater efficacy of freshwater additions over the Irminger basin in decelerating the AMOC compared to direct injections into the Labrador Sea. In the other experiments, significant shoaling of the mixed layer depth is observed (Fig. 2, B to D), with the least pronounced reduction found in HNA (Fig. 2D). For HNS, the added freshwater effectively eliminates the mixed layer depth site over the Nordic Seas present in the control simulation (Fig. 1C). Notably, the suppression of the convection sites is compensated by a generally increase in mixed layer depth across the subtropical North Atlantic and the Mediterranean Sea.

**Fig. 4.**
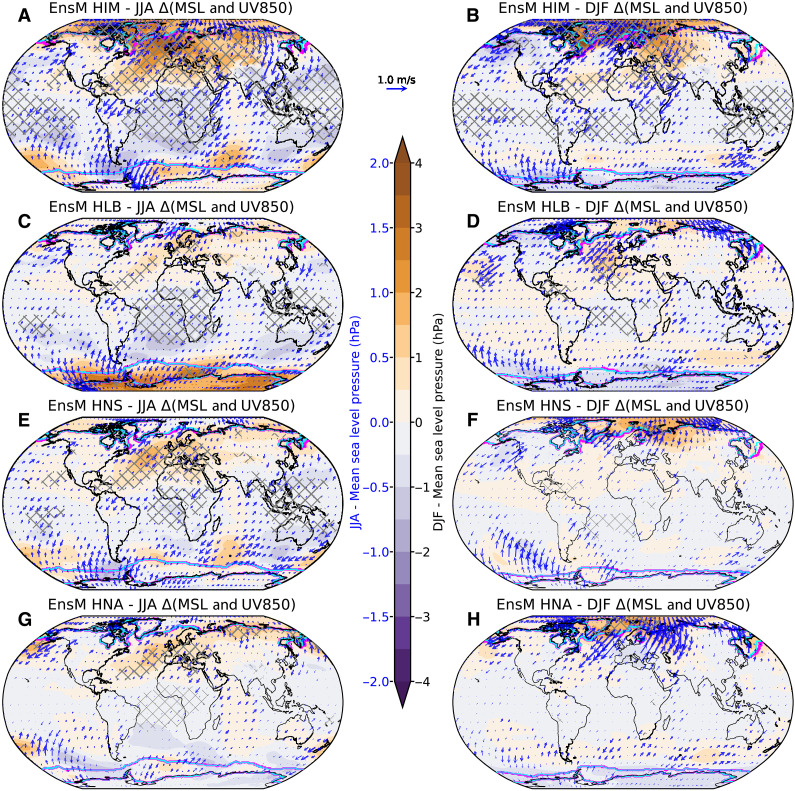
Responses of global climate to the weakened AMOC across water-hosing experiments. Shown for (**A** and **B**) HIM, (**C** and **D**) HLB, (**E** and **F**) HNS, and (**G** and **H**) HNA. The variables are displayed as the difference between the ensemble mean of each water-hosing experiment during the strongest decline period of the AMOC and the control experiment for boreal summer [June, July, and August (JJA)] and DJF. Color shadings represent the mean sea level (MSL) pressure, and blue arrows indicate wind vectors at 850 hPa. Cross-hatched areas denote statistically significant differences based on a Student’s *t* test at the 99% significance level for MSL. The contours indicate the sea ice extent, with cyan lines for the control experiment and magenta lines for the water-hosing experiment. The sea ice edge is defined as a sea-ice thickness greater than 0.05 m.

The response of the AMOC to freshwater perturbations depends not only on the original location of deep water formation regions but also on the routes through which the hosing water circulates (*24*). Reorganization of seasonal atmospheric circulation and changes in surface wind patterns can play crucial roles in redistributing freshwater, thereby affecting the mixed layer depth across different regions. For HIM, a significant increase in mean sea level pressure over the northern high latitudes, along with a noticeable reduction in surface westerlies south of Greenland, is observed (Fig. 4). Instead, this pattern is characterized by enhanced northeasterly winds, particularly in DJF, which directs freshwater from the Irminger Basin toward the Labrador Sea, contributing to the significant decline in mixed layer depth there (Fig. 2A). This is consistent with a recent modeling study Zhu and Cheng (*48*), which suggests the strong vulnerability of the Labrador Sea to external forcing, likely due to the weak ocean stratification in that region. For HLB, intensified mean sea level pressure is observed, especially over the subpolar gyre region (Fig. 4, C and D), facilitating stronger southwesterlies that help transport freshwater to the southern Irminger basin (Fig. 2B), leading to a significant shoaling of the mixed layer. In HNS and HNA, changes in atmospheric circulation and wind patterns fail to effectively channel freshwater toward key areas of deep water formation, resulting in limited impacts on AMOC strength (Fig. 4, E to H).

Although our simulations suggest that the Labrador Sea is more vulnerable than the Irminger Basin (Fig. 2A), direct freshwater input into this region appears less effective at driving an AMOC slowdown. Beyond the wind patterns responses, this discrepancy may arise from the differing contributions of water masses to AMOC formation in these two subpolar regions. Observations indicate that deep water formation in the Labrador Sea may not prominently influence AMOC variability in the subpolar basin (*49*). Analysis of AMOC within a density framework, which measures its changes due to variations in surface water mass transformations, suggests that the conversion of northward warm and salty waters into southward colder and fresher deep waters in the Irminger and Iceland basins plays a major role in subpolar AMOC variability. This finding is further supported by recent modeling studies (*34*, *50*). Notably, using the same ocean model [Finite-Volume Sea ice Ocean Model (FESOM)] as in our study, Sidorenko *et al.* (*50*) suggested that the waters contributing to the upper limb of the AMOC, where its maximum is located, are primarily formed in the Irminger Sea, with the Labrador Sea contributing to a lesser extent. Conversely, waters from the Labrador Sea largely determine the lower part of the AMOC mid-depth cell, part of which contributes to the recirculation cell.

Our findings highlight that the Irminger Basin is particularly effective to freshwater forcings that weaken the AMOC, while the North-East Atlantic demonstrates a lesser degree of sensitivity than other sites of deep water formation regions. The Labrador and Nordic Seas, while similarly sensitive to freshwater disturbances, exhibit distinct mechanisms leading to AMOC weakening. In the Labrador Sea, the reduction in AMOC strength appears to stem from both the shallowing of the mixed layer depth and alterations in atmospheric circulation and wind patterns. Conversely, in the Nordic Seas, the primary factor is the total halt of convection processes.

### Suppressed and compensated MHT

Throughout all water-hosing experiments, there is a notable decrease in global MHT during the strongest decline period of AMOC (Fig. 3B). Substantial reductions in MHT span from the tropics to the extratropical areas of the NH, with the largest decrease found in HIM, where the reduction is approximately twice that of the other three experiments. The decline in MHT in the North Atlantic basin predominantly contributes to the global MHT reduction (Fig. 3B). However, the Indo-Pacific Ocean displays positive MHT anomalies, suggesting an enhanced northward heat transfer from the Indo-Pacific Ocean basins across the equator to the NH high latitudes. This compensates, in part, for the reduced northward heat transport in the Atlantic, reflecting interbasin MHT compensation under a weakened AMOC, consistent with previous findings from water-hosing experiments in paleoclimate contexts (*51*). The enhanced MHT is larger in HIM than in the other experiments, corresponding to the greatest decline in AMOC strength (Fig. 3A), underscoring a considerable climate impact, predominantly in the NH.

The different responses of the MHT over various ocean basins reflect the interior oceanic pathways connecting the Atlantic and Indo-Pacific Oceans (*52*–*54*). Further decomposition reveals that the enhanced MHT in the Indo-Pacific Basin is mainly due to increased MHT over the Indian Ocean across all water-hosing experiments (fig. S4), largely due to the import heat from the Southern Ocean (*55*). We attribute the anomalous heat transport in the Indian Ocean to changes in circulation and temperature, following (*55*). Both factors contribute to the enhanced MHT over the Indian Ocean; however, circulation-driven heat transport anomalies play a dominant role in this process (text S2 and fig. S4).

We next examine the vertical structure of temperature evolution over the Indian Ocean (Fig. 3C). In response to the weakening AMOC, a consistent warming signal appears for all experiments, characterized by stronger subsurface warming anomalies that gradually intrude into the ocean surface and deep ocean. This warming signal can persist for a long time, even after the AMOC strength recovers. During the strongest decline period of the AMOC, the warming anomalies tend to penetrate the ocean surface and propagate quickly into the deep ocean. This indicates that interior ocean heat exchanges play a crucial role in compensating for the suppressed global MHT. This process partially facilitated the strengthening of subsurface warming in the Indian Ocean, primarily driven by oceanic circulation changes (fig. S4). The increased MHT changes over the Indian Ocean also contributes to heat exports to the Pacific via the weakened Indonesian Throughflow, consistent with the findings of Li and Liu (*55*), contributing to warming in the Pacific basin.

The Indian Ocean warming induced by the weakened AMOC may diverge from previous studies, such as (*54*), which proposed that Indian Ocean warming could enhance AMOC strength by increasing the northward transport of ocean salinity and through atmospheric teleconnections. This discrepancy emphasizes the importance of changes in interbasin connections, not only at the ocean surface but also through interior ocean exchanges, supporting the concept of a thermal interbasin seesaw between the North Atlantic and the subsurface Indo-Pacific (*53*, *55*).

### Contrast hemispheric response of surface temperature

**Fig. 5.**
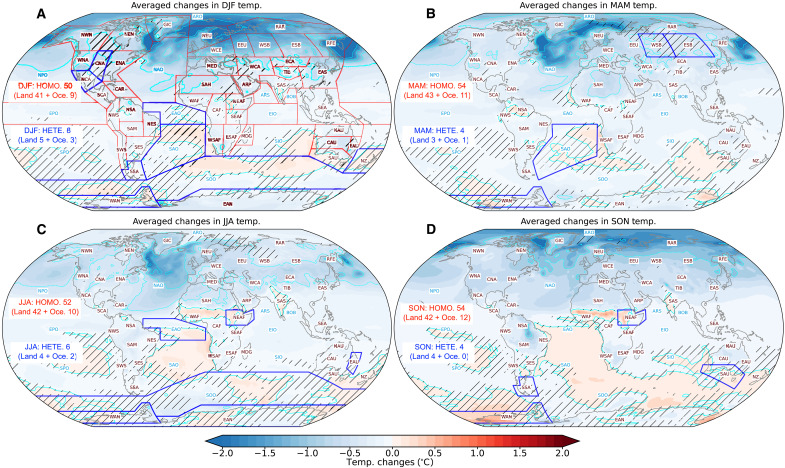
Homogeneity and heterogeneity of surface temperature response. Seasonal response of surface air temperature anomalies averaged among the four water-hosing experiments: (**A**) DJF, (**B**) spring (MAM), (**C**) JJA, and (**D**) autumn (SON). The anomalies for each water-hosing experiment are calculated as the difference between the surface air temperatures during the strongest decline period of the AMOC and the control experiment. Hatched shading (absence of shading) regions indicate heterogeneity (and homogeneity) of temperature changes, signifying differing (similar) warming or cooling responses among the water-hosing experiments. Annotated texts represent the acronyms of each IPCC AR6 reference region (details available in text S3 and fig. S5). Regions outlined in blue denote those with heterogeneous seasonal temperature changes across the water-hosing experiments. Red and blue texts indicate the number of subcontinental regions presenting homogeneous (HOMO) and heterogeneous (HETE) changes, respectively, while the numbers in brackets show the count of affected land regions and ocean basins.

The conspicuous reduction in northward heat transport is intricately linked to a significant cooling across the majority of the NH (Fig. 5, fig. S5A, and fig. S6). In contrast, the SH exhibits a slight warming. This observed contrast hemispheric surface temperature response aligns generally with previous studies proposing the “bipolar seesaw pattern” in responses to a slowdown or collapse of the AMOC, especially in experiments involving extensive freshwater hosing over the North Atlantic (*2*, *3*, *18*, *56*, *57*). However, our results reveal that the distinct bipolar seesaw responses in surface temperature may depend on the season and the freshwater injection regions. For example, during June, July, and August (JJA), the bipolar seesaw responses is relatively evident in HIM (fig. S6B) and HLB (fig. S6D) but not in other experiments.

Surface temperature presents regional variations in response to a weakened AMOC, previously overlooked. To evaluate both homogeneous and heterogeneous temperature changes at a subcontinental level, our study uses the latest reference regions defined by the IPCC AR6 [text S3 and fig. S5; (*58*)]. Our comparison of temperature variability across seasons within four different experiments reveals that 86 to 93% of the subcontinental regions undergo homogeneous temperature changes, irrespective of the season (Fig. 5). The most dominant cooling occurs in DJF, particularly over the North Atlantic (Fig. 5A), coinciding with the suppressed ocean deep convection (Fig. 2). Warm anomalies predominantly spread over the oceanic regions in the SH, although temperature changes show more heterogeneity, especially in the South Atlantic and Antarctic.

Heterogeneous temperature responses tend to increase slightly during DJF and JJA (Fig. 5,A and C) compared to March, April, and May (MAM) and September, October, and November (SON) (Fig. 5, B and D), likely due to the diverse local effects of different dynamic and physical processes. In the detailed examination of temperature responses during DJF and JJA (fig. S6), heterogeneous temperature changes are observed in some places. Central North America exhibits a notable warming anomaly in DJF in HLB while showing a general cooling in the other experiments. This heterogeneous temperature change explains the heterogeneity of temperature responses in Fig. 5A over North America. Moreover, although the cooling is broadly consistent, the intensity of local cooling varies with the freshwater flux injection sites. As in JJA, the HIM induces the most significant cooling across NH land surfaces and in regions with significant deep convection reduction, where the maximum cooling can reach 4° to 6°C (fig. S6A). Other experiments (fig. S6, C, E, and G) show cooling confined to high-latitude land surfaces of northern Eurasia. A noticeable heterogeneous pattern emerges from the Barents Sea to central Eurasia due to a significant warming belt identified in HNA (fig. S6H). In addition, anomalous warming over parts of India and the West African monsoon regions suggests the influence of a weakened AMOC on the global summer monsoon (*59*).

Associated with the suppressed northward heat transport, a weakened AMOC contribute to the expansion of Arctic sea ice (Fig. 4 and fig. S7), consistent with findings from (*28*). The sea ice expansion may be linked to widespread high-latitude cooling over the NH through the ice-albedo feedback mechanism (*2*). In addition, the reduction or cessation of deep convection in the North Atlantic (Fig. 2) could also contribute to sea surface cooling, inhibiting heat transfer from the interior ocean to its surface (*45*). Furthermore, the observed cooling extending across both oceanic and land surfaces may also be propelled by other mechanisms, including high-latitude temperature advection facilitated by alterations in atmospheric circulation (Fig. 4 and figs. S8 and S9) and the interplay of wind-evaporation–sea surface temperature feedbacks (*60*). However, the heterogeneous warming signal from HNA during JJA (fig. S6H) could be linked to the regional retreat of sea ice extent (Fig. 4G and fig. S7), facilitating positive ice-albedo feedback and influencing the anomalous anticyclone system from the Barents Sea to central Eurasia (fig. S9).

Our analysis of the impacts of freshwater inputs from different regions on surface temperatures reveals a complex landscape. We argue that the generally bipolar seesaw pattern in surface temperatures response to a slowdown AMOC seems more overestimated and may depend on the varying locations of freshwater perturbations. The effect on land surface temperatures exhibits a more localized nature. At high latitudes, the ice-albedo feedback mechanism plays a pivotal role in driving temperature variations across local and neighboring land areas. Changes in atmospheric circulation could either intensify or spread these temperature anomalies further. Moreover, an interesting pattern of a consistent warming is observed over India and the southern Sahara during warmer seasons. This warming in India might be tied to a slight increase in MHT within the Indo-Pacific tropics (Fig. 3, B and C, and fig. S4), while the warming in the southern Sahara could relate to a southward shift of the ITCZ (*2*).

### Homogeneity and heterogeneity precipitation changes

**Fig. 6.**
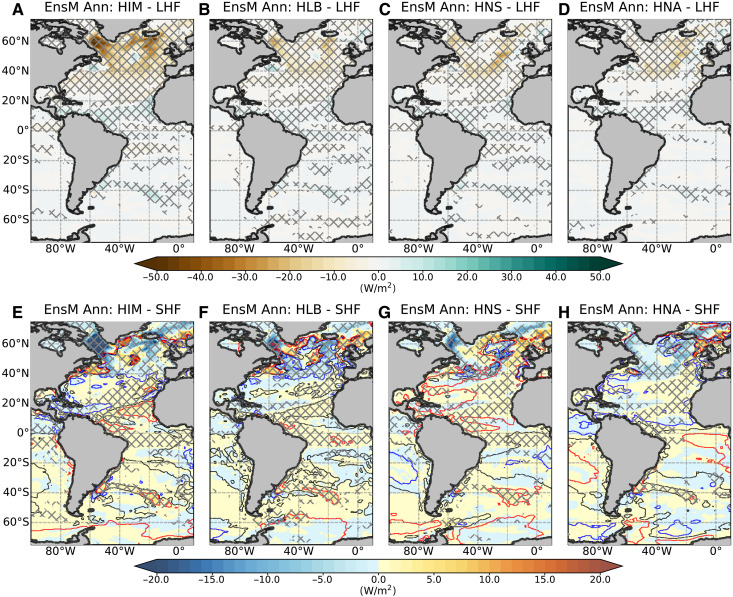
Response of annual surface turbulent heat fluxes under weakened AMOC over the Atlantic. Differences in (**A** to **D**) latent heat flux (LHF) and (**E** to **H**) sensible heat flux (SHF) for different water-hosing experiments during the strongest decline period of AMOC compared to the control experiment. Cross-hatched areas denote statistically significant differences based on a Student’s *t* test at the 99% significance level. Contour lines in (E) to (H) show the difference between annual sea surface temperature and surface air temperature anomalies. Red, gray, and blue lines indicate where the former is warmer, equal to, or colder than the latter by 0.1°, 0°, and −0.1°C, respectively.

In response to a weakened AMOC, changes in atmospheric circulation and surface temperature can modify air-sea heat fluxes. We focus on the changes in annual surface turbulent heat fluxes, including latent and sensible heat fluxes (Fig. 6). The surface turbulent heat flux shows significant negative anomalies, especially over the northern high latitude North Atlantic for HIM. This is mainly due to the significant decrease of surface temperature (Fig. 5 and fig. S6), changing wind patterns from westerlies to northeasterlies, and the southward extension of sea ice (Fig. 4 and fig. S7), which together suppress ocean-atmosphere thermal coupling. Slightly positive anomalies in sensible heat flux over the subtropical North Atlantic are linked to a slightly strengthening of the subtropical gyre, as indicated by positive anomalies in the mean sea level pressure and wind patterns (Fig. 4). In addition, annual sea surface temperature anomalies in this region are generally warmer than surface air temperatures (Fig. 6, E to H), suggesting that the strengthened gyre and warmer sea surface may partly offset total turbulent heat loss. Moreover, changes in surface turbulent heat fluxes are closely related to the alternations in precipitation. Substantial changes in global precipitation patterns are observed, accompanied by suppressed surface turbulent heat fluxes and atmospheric eddy moisture transport (*3*, *61*).

**Fig. 7.**
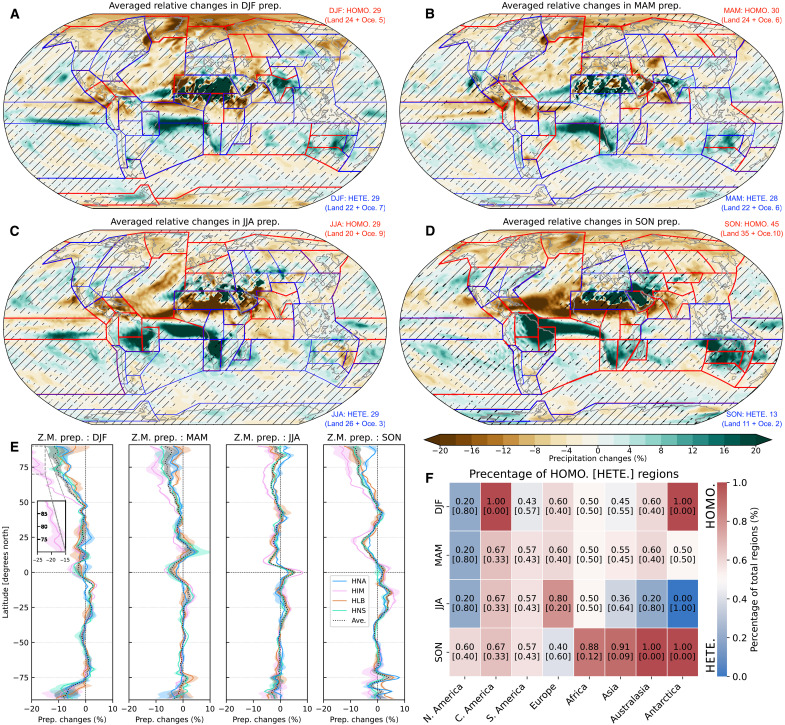
Homogeneity and heterogeneity of seasonal precipitation changes under weakened AMOC. The relative changes in seasonal precipitation in comparison to control experiments across various seasons: (**A**) DJF, (**B**) MAM, (**C**) JJA, and (**D**) SON. Hatched shading (absence of shading) regions indicate heterogeneity (and homogeneity) of precipitation changes, signifying differing (similar) drying or wetting responses among the water-hosing experiments. Boundaries outlined in red (blue) delineate regions exhibiting homogeneous (heterogeneous) precipitation changes within each IPCC AR6 reference region. (**E**) Illustrates the zonal mean (Z.M.) of precipitation change for each water-hosing experiment, with the dotted line showing the aggregate average of these changes. (**F**) Summaries by continent of the percentage of subregions exhibiting homogeneous and heterogeneous precipitation changes across the global land surface (details available in text S4 and figs. S11 to S16).

Seasonal precipitation changes mirror those in surface temperature and latent heat flux, exhibiting a broadly homogeneous hemispheric contrast (Fig. 7, A to E), with drying in the NH and slight wetting in the SH. The NH, especially high-latitude regions, experiences widespread decreases in precipitation, consistent with surface cooling and reduced atmospheric moisture. Notably, the North Atlantic and Gulf Stream show significant decreases in precipitation across all seasons and experiments, especially during DJF and JJA. Conversely, the SH witnesses increased precipitation, particularly over the Amazon basin during JJA and SON, aligning with discussions suggesting that a weakened AMOC could delay or prevent Amazon rainforest dieback (*62*).

Zonal mean changes in seasonal precipitation signal a consistent southward shift of the ITCZ (Fig. 7E and fig. S10), with decreased precipitation in its northern stretches and increased precipitation to the south. Previous studies have linked this southward migration to variations in hemispheric surface temperature gradients (*2*, *61*), changes in atmospheric energetics (*3*), and the impact of strengthened anomalous trade winds (*63*). Consequently, this shift has led to pronounced changes in seasonal precipitation patterns across the tropical Atlantic and global monsoon regions, further influenced by the induced anomalous trade winds (figs. S8 and S9).

The observed homogeneous precipitation changes underscores the consistent impact of a weakened AMOC, while the diverse outcomes across water-hosing experiments (hatched areas in Fig. 7) highlight the importance of acknowledging heterogeneity. This heterogeneity necessitates comprehensive investigation, especially considering potential societal impacts. We then investigate seasonal precipitation changes at the subcontinental scale, providing detailed insights into the interaction between a weakened AMOC and localized precipitation patterns. The examination of regional precipitation variations across different water-hosing experiments is thoroughly examined for all IPCC AR6–defined regions (Fig. 7F and figs. S11 to S16). Briefly, 45 of 58 (35 land and 10 ocean basins) show homogeneous changes during SON (Fig. 7D). In contrast, boreal winter (Fig. 7A) and summer (Fig. 7C) exhibit more variability, with 29 regions showing heterogeneous responses. Most land regions during SON and MAM show generally homogeneous precipitation changes. Heterogeneous precipitation changes across North America are notable for most seasons, highlighting the regional vulnerability to complex precipitation changes under a weakened AMOC.

Specifically, northern high-latitude regions, such as Greenland/Iceland and Northern Europe, consistently show reduced precipitation year-round. The tropics display uniform reduction in some monsoon areas, such as Central America and the Caribbean, with increase in northern South America and West Southern Africa. The Sahara and India tend to dry out in warmer seasons and become wetter in colder ones. Mid-latitude regions exhibit predominantly heterogeneous precipitation changes, influenced by seasonal variations and specific water-hosing experiments. Ocean basins largely show homogeneous changes, with precipitation decreasing in the northern oceans and increasing in the tropical Atlantic. The magnitude of these changes depends on the specific water-hosing experiment, with HIM demonstrating the most pronounced seasonal variations, followed by HNS and HLB, and HNA showing the lease impact, reflecting the diverse intensity of AMOC reduction across these experiments (Fig. 3).

In summary, large-scale seasonal changes in precipitation show homogeneity, consistent with earlier studies (*18*, *20*, *45*, *57*). There is a pronounced reduction in precipitation from the northern mid-latitudes to high latitudes and an increase from the deep tropics to the southern subtropics. However, our results suggest that precipitation patterns within both northern and southern mid-latitude areas exhibit considerable heterogeneity. This variability likely stems from complex interactions within atmospheric circulation changes and the shift of ITCZ induced by the weakened AMOC, further influenced by diverse freshwater sources and seasonal variations.

### Implications for temperature and precipitation extremes

**Fig. 8.**
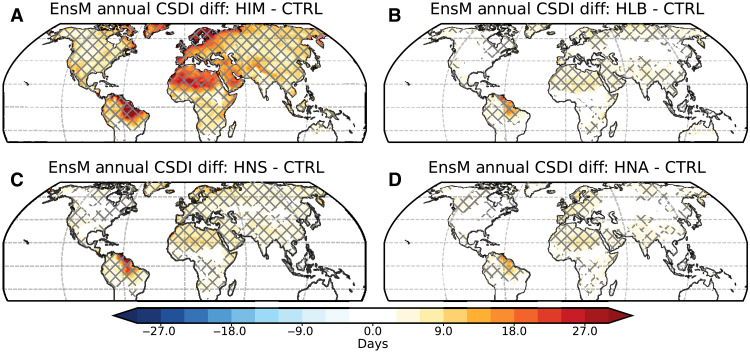
Changes in cold extremes under weakened AMOC in different water-hosing experiments. Cold extremes are measured by the CSDI and shown as the difference between the strongest decline period of AMOC and the control experiment: (**A**) HIM, (**B**) HLB, (**C**) HNS, and (**D**) HNA. Cross-hatched areas denote statistically significant differences based on a Student’s *t* test at the 99% significance level.

So far, we have investigated the climatic changes induced by the weakened AMOC in response to freshwater input from different regions. These changes could substantially affect regional extreme weather events, depending on the varying regional weather responses (*64*–*66*). Given the examined widespread cooling induced by the weakened AMOC, we first investigate its impacts on cold extremes over the global land surface (Fig. 8). In response to general cooling, the cold spell duration index (CSDI; Materials and Methods) presents a broadly increasing pattern. The magnitude of this response depends on the extent of AMOC weakening, which is determined by the freshwater injection regions. Nevertheless, a homogeneous pattern is observed, with an increase in cold spell duration over northern Europe, northern South America, and the Sahara region. Notably, except for HLB, Europe experiences a significant increase in cold spell duration in all other experiments. The result for HLB aligns with (*65*), which reveals a decrease in cold spells over Europe, whereas the other three experiments indicate an inconsistent pattern. This inconsistency highlights the importance of identifying key regions in driving AMOC variations, particularly in studying regional manifestations of temperature extremes.

**Fig. 9.**
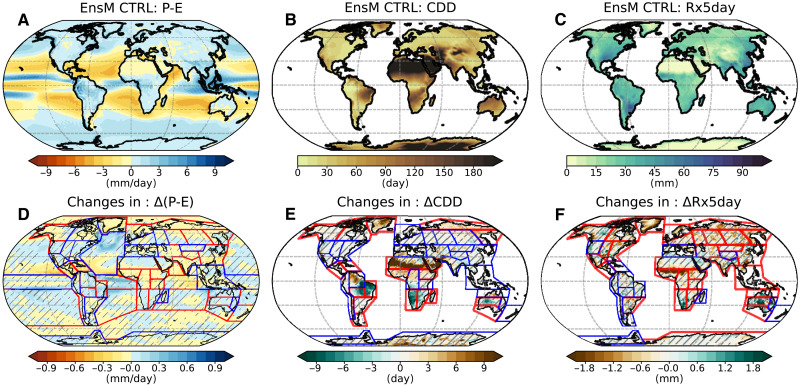
Climatology and changes in the hydrological cycle and precipitation extremes. Climatology of (**A**) total annual precipitation minus evaporation (P-E), (**B**) dry spell length (CDD), and (**C**) heavy precipitation intensity (RX5day) in the control simulation. (**D** to **F**) Homogeneous and heterogeneous changes in P-E, CDD, and RX5day during the strongest decline period of AMOC.

Heterogeneity in changes induced by different water-hosing experiments also exists in the global hydrological cycle and precipitation extremes (Fig. 9). The hydrological cycle is measured by annual precipitation minus evaporation (P-E), which presents an almost identical climatological pattern (Fig. 9A) to the observations in (*67*). Homogeneous and heterogeneous changes in P-E reflect changes in global precipitation patterns (Fig. 7), resulting in more heterogeneity over mid-latitude and monsoon regions (Fig. 9D). The largest changes prevail over the North Atlantic Ocean, resulting from cooling-suppressed evaporation. Regarding precipitation extremes, we consider the dry spell index [consecutive dry day (CDD); Fig. 9, B and E] and the heavy precipitation intensity index (RX5day; Fig. 9, C and F). Across different water-hosing experiments, more regional heterogeneity is observed for CDD over northern mid-latitude and South Asian monsoon regions. Although there is a homogeneous decrease in CDD around the Amazon Basin, the heavy precipitation intensity index shows heterogeneous changes there. Our results suggest that precipitation changes induced by a weakened AMOC could lead to varying regional occurrences of drought and flood events, depending on the regions of freshwater release over the North Atlantic.

## DISCUSSION

Our study investigates the sensitivity of AMOC slowdown to varying freshwater fluxes from different North Atlantic regions, motivated by growing concerns about the projected AMOC slowdown and potential collapse due to anthropogenic global warming (*68*, *69*). Findings suggest that continuous freshwater fluxes into the Irminger basin exert the most pronounced effect on AMOC weakening, aligning with recent observational studies (*34*, *36*), highlighting the crucial role of the Irminger Sea in modulating AMOC variability. In our water-hosing experiment over the Irminger Sea, we observe a near halt in convection across the Labrador Sea and the Nordic Seas, linked to intensified northeast winds over West Greenland and stronger southwest winds around the eastern subpolar gyre. These conditions likely facilitate the transport of freshwater, inhibiting deep water formation and consequently weakening AMOC strength. The anomalous wind and atmospheric circulation patterns resemble a positive North Atlantic Oscillation–like pattern, contributing to spreading freshwater from the Irminger Sea to the Labrador Sea and Nordic Seas via the subpolar gyre (*70*–*72*).

We explore the homogeneous and heterogeneous regional climate impacts of a weakened AMOC due to freshwater fluxes from different areas. The large-scale homogeneous climate impacts show a general independence from the regions of freshwater introduction. Notably, climatic impacts are more pronounced when freshwater is added to the Irminger Sea. Across all seasons, we observe consistent NH cooling and SH slight warming, primarily due to reduced northward heat transport in the Atlantic. The cooling is more intense where convection is largely reduced, coinciding with increased sea ice concentration, while mild warming is seen over the South Atlantic and Southern Indian Ocean. This is related to the general increased oceanic heat transport across the Indo-Pacific Ocean, compensating for suppressed northward heat transport over the Atlantic. In addition, this altered interhemispheric temperature gradient leads to decreased (increased) precipitation in the NH (SH), particularly along the Gulf Stream in summer. Furthermore, the AMOC slowdown induces a southward shift of the ITCZ, substantially affecting precipitation in the tropical Atlantic and global monsoon regions.

Homogeneous temperature responses under a weakened AMOC are intricately linked to sea ice responses via ice-albedo feedback in high latitudes and more related to atmospheric dynamics and changes in oceanic heat transport in mid-latitudes. However, we emphasize the importance of exploring the heterogeneous regional climate impacts of AMOC slowdown, a critical aspect often overlooked in research focused on global or European contexts (*18*, *45*). We particularly highlight the heterogeneous effects of AMOC weakening on seasonal precipitation variations at a subcontinental scale, such as in North America. This heterogeneity underscores the complexity of changes across both land and ocean basins. We propose that the southward ITCZ shift and changes in surface turbulent heat fluxes are major mechanism behind disparate regional precipitation patterns. Changes in ITCZ location not only alter monsoon rainfall patterns but also affect atmospheric circulation shifts (*71*, *73*) and global teleconnection patterns (*2*). Beyond the mean climate responses, temperature and precipitation extremes in northern mid-latitude regions exhibit pronounced heterogeneity, particularly in the persistence of extreme events.

Our study uses a single climate model under pre-industrial conditions, complemented by a limited ensemble revealing robust patterns. While valuable, these insights should not serve as direct predictions of future climate scenarios. We recommend future investigations under enhanced climate warming conditions (*3*, *38*) to better understand the unique responses of a weakening AMOC to greenhouse gases and anthropogenic aerosols (*5*). We applied an idealized freshwater volume to induce AMOC weakening, acknowledging that different regions and durations of water-hosing could elicit varied regional climate responses. It remains uncertain whether consistent climate responses would be observed when equilibrium is reached following freshwater injection into different regions. Given the lack of agreement among experts regarding vulnerable regions to heightened freshwater influxes affecting AMOC (*39*, *74*, *75*), additional simulations using other climate models are imperative.

The primary objective of our study is to establish a foundation and present one possibility for identifying the regional climatic responses of a weakened AMOC, emphasizing the importance of examining regions susceptible to inducing an AMOC slowdown. While large-scale climate responses to a weakened AMOC are generally consistent across models, a key limitation of our study is that homogeneous and heterogeneous climate responses at the subcontinental scale are highly model dependent, particularly with respect to hydrological changes. Factors such as atmospheric parameterization schemes, model resolution, and the sensitivity of deep-water formation regions to water-hosing are critical in assessing subcontinental climate responses (*29*, *48*, *76*, *77*). Therefore, future research must engage a broader climate modeling community. Sensitivity analyses within the North Atlantic Hosing Model Intercomparison Project (NAHosMIP) project framework (*23*) could serve as a valuable next step in this effort.

Overall, our findings provide critical perspectives on past, present, and future climate changes related to AMOC variations. We emphasize that different freshwater sources and injection regions can trigger substantial heterogeneous regional climate responses, particularly in the context of regional weather extremes. In light of these results, we advocate for expanded observational and modeling studies focused on the Irminger Basin (*4*), as this region may play a critical role in understanding the dynamic mechanisms and predicting the future strength of the AMOC.

## MATERIALS AND METHODS

### Global climate model

In this study, the global climate model used is the new version of the AWI-CM3, a fully coupled model comprising the atmosphere and ocean (*35*). In particular, the atmosphere model (OpenIFS) is based on the global Integrated Forecasting System in version 43R3V1, developed at the European Centre for Medium-Range Weather Forecast (ECMWF). We conduct simulations at a resolution of Tco95 (∼100 km) with 91 vertical layers (TCo95L91). The ocean model used is the FESOM2, a global unstructured-mesh ocean model developed at AWI (*78*, *79*). The unstructured mesh of FESOM2 contains around 127,000 surface nodes (*80*). It comprises 47 vertical layers, with horizontal resolution varying from 25 to 125 km. Refined resolution regions are set in high latitudes, the equator, and coastal areas. The sea ice component is embedded in FESOM2 and discretized and integrated on the same unstructured grid as the ocean model. The model components iterate via a concurrent coupling strategy that allows the exchange of surface heat, mass, and momentum fluxes between the atmospheric and oceanic components.

The AWI-CM3 has been shown to perform better than the average of the CMIP6 models in representing global climatology (*35*). The stand-alone FESOM2 has been validated for its ability to simulate a realistic AMOC and has also demonstrated an improvement in the representation of the Gulf Stream and North Atlantic Current (*79*). Sidorenko *et al.* (*80*) reported that the coupled FESOM2 and OpenIFS exhibits well performance in representing global-mean surface heat and freshwater budgets, as well as ocean hydrography. Therefore, the above evidence demonstrates the ability of the new climate model, AWI-CM3, to simulate the variability of AMOC and its far-reaching global influences.

### Experimental design

To investigate the impact of a slowdown AMOC, we weaken the AMOC strength by artificially releasing freshwater into the North Atlantic region using a similar experimental design following previous studies (*2*, *18*, *23*). These freshwater perturbation experiments, commonly referred to as water-hosing experiments, are based on the pre-industrial control experiment. The PI-CTR experiment runs for 200 years, which is branched off from a 700-year-long spin-up, as shown in (*35*). External forcings, such as greenhouse gases and solar forcing, are fixed at the year 1850. To account for internal variability, a small three-member ensemble has been conducted. The other two members are initialized from 1 and 10 years off the first control run. This small ensemble approach helps reveal robust features by using different initial conditions.

As for the water-hosing experiments, Jackson *et al.* (*23*) proposed an experimental framework, NAHosMIP, which designed water-hosing experiments by imposing additional freshwater input over the North Atlantic and along the coastal region around Greenland. This framework covers a border region supplied the freshwater fluxes to the ocean, while the sensitive regions responsible for changing AMOC are uncertain. The strength of the AMOC is sensitive not only to the regions where deep water formation take place but also to regions where there are changes in the net freshwater flux along the AMOC route (*45*). To explore the impact of water-hosing experiments in the North Atlantic, we define four distinct regions: the Irminger basin (HIM), Labrador Sea (HLB), Nordic seas (HNS), and North-East Atlantic (HNA). A detailed summary of these four regions can be found in table S1. We chose these regions based on a similar approach as in (*34*), where they assessed the local contributions to decadal AMOC variability. Although idealized, these regions have got much attention from both paleo- and modern observational perspectives, as they play major roles in driving subpolar AMOC variability (*6*, *10*, *31*, *33*, *45*, *49*).

Therefore, we designed our water-hosing experiment by inputting additional freshwater over these regions to reveal the sensitivity of climate responses to the location of freshwater forcing. The volume compensation is used to conserve global volume mean salinity (*81*). Furthermore, recent observational evidence suggests that the Irminger Sea plays a vital role in driving subpolar AMOC variability at multiple timescales (*34*, *36*, *74*); however, few climate models have supported these observations. Our sensitivity experiments can therefore offer insights into relating to the observations.

Similar to the PI-CTR, we conduct three ensemble members for each regional water-hosing experiment to accentuate the signal and ensure robust responses. Each water-hosing experiment starts with the same initial climate state as the corresponding ensemble member from the PI-CTR experiments. The freshwater hosing strength remains consistently set at 0.1 Sv (1 Sv = 10^6^ m^3^ s^−1^) across the four defined regions, normalized to the covered oceanic grids. This ensures that the total freshwater flux is identical across all experiments. We note a caveat to this approach is the differing ocean areas, representing a compromise in our examination of the sensitivity of AMOC to hosing regions. In addition, although this hosing strength is idealized, it is considered a reasonable approach for reducing the AMOC strength (*6*, *23*, *45*, *82*), and it represents a substantial estimate of potential freshwater input from melting glaciers in Greenland (*22*, *23*). We introduce freshwater continuously at a rate of 0.1 Sv for the first 50 years (a total of 5 Sv over 50 years), followed by recovery experiments where no hosing is applied, allowing integration to continue for another 150 years.

To mitigate numerical challenges, such as abrupt freshwater gradient changes, at the regional boundaries surrounding hosed regions, we generate the hosing field FWh for the water-hosing experiments. A hosing field, corresponding to a given hosing strength hSv, can be formulated as follows

$$F\_\text{lat}\left(x\right)=\left\{\begin{array}{cc} \text{cos}\left(\mathrm{kx}\right) & |x|<L,k=\pi /2L\\ 0 & |x|>L \end{array}\right.$$ (1)

$$F\_\text{lon}\left(y\right)=\text{exp}\left(-0.01*y^{2}\right)$$ (2)

FWh=hSv×F_lat(x)×F_lon(y) (3)

where *L* represents the zonal range for each region, and in our study, hSv is set to 0.1 Sv. The lat and lon are the zonal and meridional boundaries of each region (table S1). The formulation of the hosing field is inspired by and adapted from Gill (*83*), where they investigated tropical circulation responses to an idealized heating field. In our approach, freshwater forcing is introduced with its maximum value at the center of the region and gradually diminishes toward the regional boundaries (Fig. 2). Note that our hosing field is exclusively applied over the oceanic grids in AWI-CM3.

### Homogeneous and heterogeneous climate responses

We use the latest reference regions defined by the IPCC AR6 to evaluate climate responses to a slowdown of the AMOC (text S3 and fig. S5). This reference framework, which encompasses 46 land and 12 ocean regions globally, provides a nuanced approach for analyzing regional climate changes (*58*). Within this framework, we assess homogeneous and heterogeneous climate responses across different water-hosing experiments on both subcontinental and seasonal scales.

To assess the homogeneity and heterogeneity of a given climate response, we first calculate the ensemble-mean area-weighted mean of the relevant climatic variable for each specific reference region in both the control and water-hosing experiments. For each water-hosing experiment and each reference region, we then compute the differences relative to the control simulation over a defined period. If the sign of the change is consistent across all water-hosing experiments for a given region, then we define the response as homogeneous; conversely, if the sign of the change in one of the water-hosing experiments differs from the others, then we define the response as heterogeneous for that region.

### Extreme indices

To explore the impacts of a slowdown of AMOC on extreme temperature and precipitation events, several extreme indices are computed according to the definitions from the Expert Team on Climate Change Detection and Indices (*84*). All indices are calculated on an annual scale (model year):

CSDI: Annual count of days when there are at least six consecutive days with daily minimum temperatures below the 10th percentile. The 10th percentile is calculated from the ensemble mean of the control simulation.

CDD: Also known as dry spell index, this is the annual count of the largest number of consecutive days with daily precipitation amounts less than 1 mm.

Heavy precipitation intensity (RX5day): The annual maximum value of consecutive 5-day precipitation amounts.

In addition, changes in the hydrological cycle induced by a weakened AMOC are estimated by measuring the changed rate of annual freshwater flux between the atmosphere and the surface (*67*). This is determined by the annual total amount of precipitation minus evaporation (P-E).

## Supplementary Materials

### This PDF file includes:

Texts S1 to S4 Figs. S1 to S16 Table S1 References
