# Supplementary material for: Revisiting climate impacts of an AMOC slowdown: dependence on freshwater locations in the North Atlantic
Source: Sci Adv. 2024 Nov 20;10(47):eadr3243. doi: 10.1126/sciadv.adr3243 (PMC11578178; doi:10.1126/sciadv.adr3243)
Supplement: Supplementary file 1 — Texts S1 to S4 Figs. S1 to S16 Table S1 References [file sciadv.adr3243_sm.pdf]

Science Advances
AAAS

Supplementary Materials for

Revisiting climate impacts of an AMOC slowdown: dependence on freshwater locations in the North Atlantic

Qiyun Ma *et al.*

Corresponding author: Qiyun Ma, qiyun.ma@awi.de

Sci. Adv. **10**, eadr3243 (2024)
DOI: 10.1126/sciadv.adr3243

This PDF file includes:

Texts S1 to S4
Figs. S1 to S16
Table S1
References

Text S1: The weakening and recovery of AMOC strength in the four water-hosing regions.

Table S1 outlines the defined areas for the introduction of freshwater fluxes across four distinct regions.

Figure S1 depicts the AMOC strength (at its maximum depth at 26.5°N) derived from areas impacted by freshwater disturbances during the initial 50 years of modeling. Across all water-hosing scenarios, a consistent decrease in AMOC strength is observed following freshwater addition to the targeted regions. The process of AMOC strength regaining its referenced level typically spans about 50 years.

Figure S2 presents the probability density functions for AMOC strength during its recuperation phase over the concluding 100 years, assuming a return to referenced conditions (illustrated by the grey line). Compared to the long-term mean from the pre-industrial control experiments, AMOC strength not only reverts to its controlled state but also exhibits an increase, particularly with freshwater input into the Irminger Sea (Fig. S2A). This enhanced AMOC strength may result from overshoot phenomena observed in these perturbation experiments (85, 86), which merits additional exploration.

This research is centered on the transient climate impacts resulting from AMOC attenuation. Thus, in the main text, we focus on AMOC changes during the first 100 years of modeling. The reference period is thus determined as the ensemble mean over the final 60 years of the initial 100 model years.

Text S2: The characteristics of AMOC in its weakest phase.

For the four experiments, Fig. S3 illustrates the ensemble mean of the differences in the meridional overturning stream function between the period of the strongest decline period of AMOC

and the reference period.

Figure S4 presents a replot of the poleward heat transport changes in the Indo-Pacific Basin, as shown in Fig. 3B of the main text. However, in this figure, the changes are further decomposed into the Indian Ocean and Pacific Ocean components. Additionally, the changes in poleward heat transport are attributed to ocean circulation-driven components (U-driven) and temperature-driven components (T-driven). The attribution methodology follows (55) and is applied during the period identified as the strongest decline of the AMOC. The main concept of this method involves calculating the meridional heat transport by using either the climatological velocity or temperature and then comparing the resulting changes with those observed during the reference period.

Text S3: The subcontinental analysis of the temperature and precipitation changes

The analysis of temperature and precipitation changes across the four water-hosing experiments is conducted at a subcontinental scale. The specific subcontinental regions are labeled and detailed in Fig. S5, following the definitions by (58). These regions align with the reference areas established for various working groups within the IPCC AR6 (16). As depicted in Fig. S5C, the reference framework encompasses 46 land and 12 ocean regions worldwide, offering a climatically coherent foundation for evaluating regional climate modifications (58). Figures S5A and S5B respectively showcase the ensemble mean of the climatological annual average temperature and precipitation from the control experiments. The spatial distributions of climatological temperature and precipitation align with the long-term average (1995-2014) derived from the CMIP6 historical experiment multi-model mean (16), further validating our examination of AMOC weakening effects on temperature and precipitation dynamics.

Figure S6 illustrates the differences in surface air temperature between the perturbed ex-

periments and the control conditions, analyzed on an winter and summer time scales. Figure S7 presents the spatial changes in summertime sea-ice concentration, while the corresponding sea-ice extent across different water-hosing experiments is shown in Fig. 4 of the main text. Figures S8 and S9 display the changes in the atmospheric circulation, demonstrated at 850 hPa, between the water-hosing and the control experiments during the strongest decline period of AMOC.

Text S4: The subcontinental analysis of precipitation changes

Figure S10 showcases the ensemble mean zonal average of seasonal precipitation in the control experiments. Subsequent figures, from Fig. S11 to Fig. S16, detail the subcontinental examination of seasonal precipitation changes relative to the local climatological mean during the strongest decline period of AMOC. Notably, the variability in precipitation response is large from region to region. For example, during winter (DJF), the reductions and increases in precipitation span from -30.44% in the North Eastern Africa (NEAF) to 118.99% in the Sahara (SAH) across various experiments (Fig. S13). Therefore, it is important to recognize that bar graphs are displayed with adjusted y-axis scales to accommodate the diverse range of changes observed in different regions.

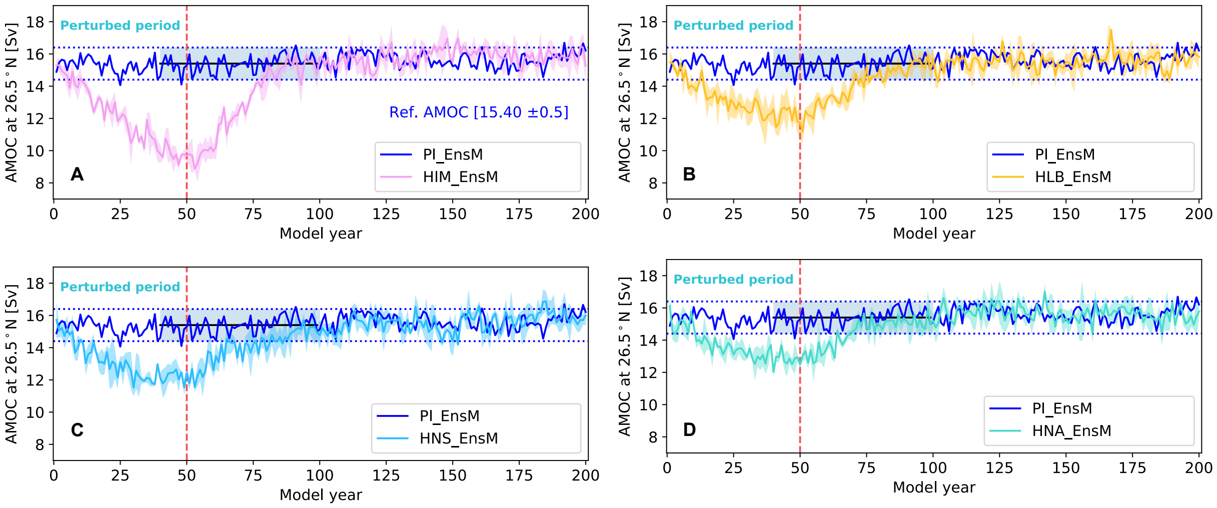

Fig. S1: **AMOC strength in control and water-hosing experiments.** Represented as indices for the four freshwater-perturbed regions (Table S1): **(A)** HIM, **(B)** HLB, **(C)** HNS, and **(D)** HNA. The vertical red dashed line indicates the conclusion of the freshwater-perturbed period (at the 50th model year), followed by the AMOC recovery period. The thick blue line denotes the ensemble mean of the AMOC index in the control experiments (PI_EnsM), while the dashed blue line represents its long-term mean $\pm$ 1σ. The blue shading indicates the selected reference period. The colored thick lines depict the ensemble mean of the AMOC index in the corresponding experiments, with shading representing the ensemble spread.

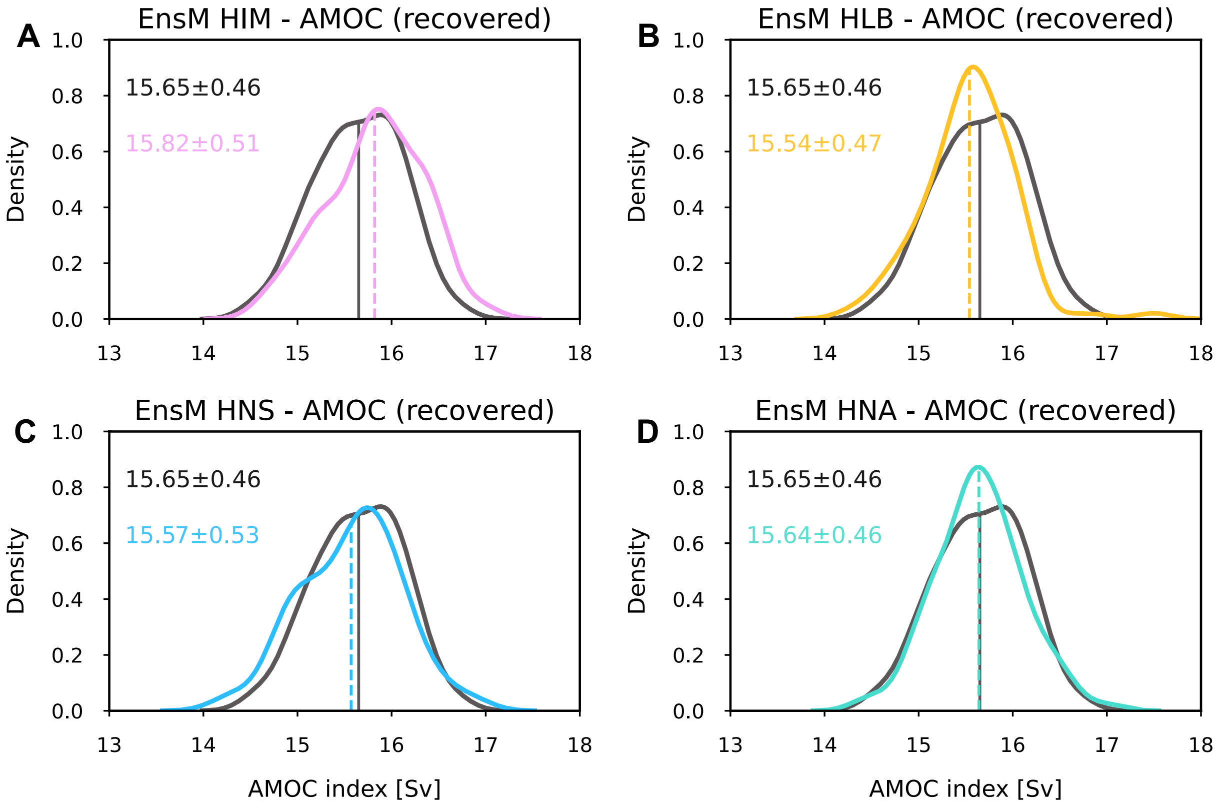

Fig. S2: **Recovered AMOC strength among the four water-hosing experiments.** Presented as probability density functions of the recovered AMOC strength (from the 100th model year onward) for: **(A)** HIM, **(B)** HLB, **(C)** HNS, and **(D)** HNA. Additional information includes the long-term mean and its corresponding one standard deviation.

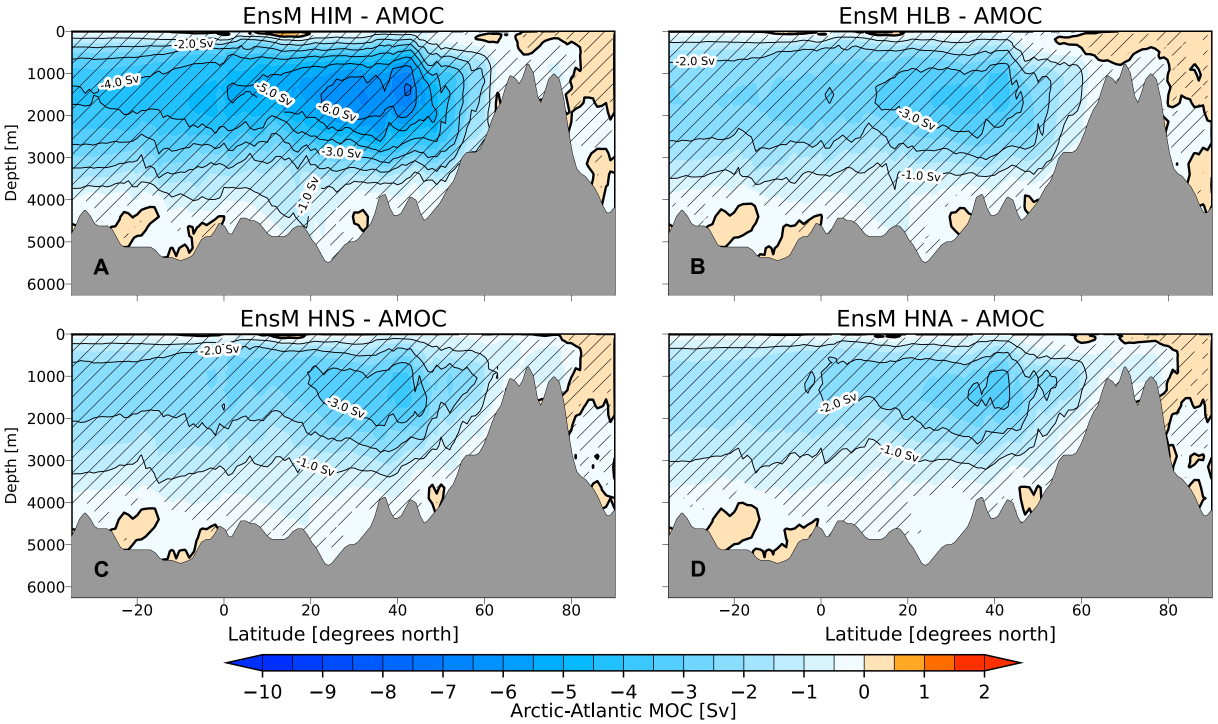

Fig. S3: **Changes in the AMOC stream function.** The AMOC stream function anomalies during the defined strongest decline period of AMOC with respect to the reference period from the control experiment. Hatched areas indicate significance at the 99% confidence level using a two-side Student's t-test.

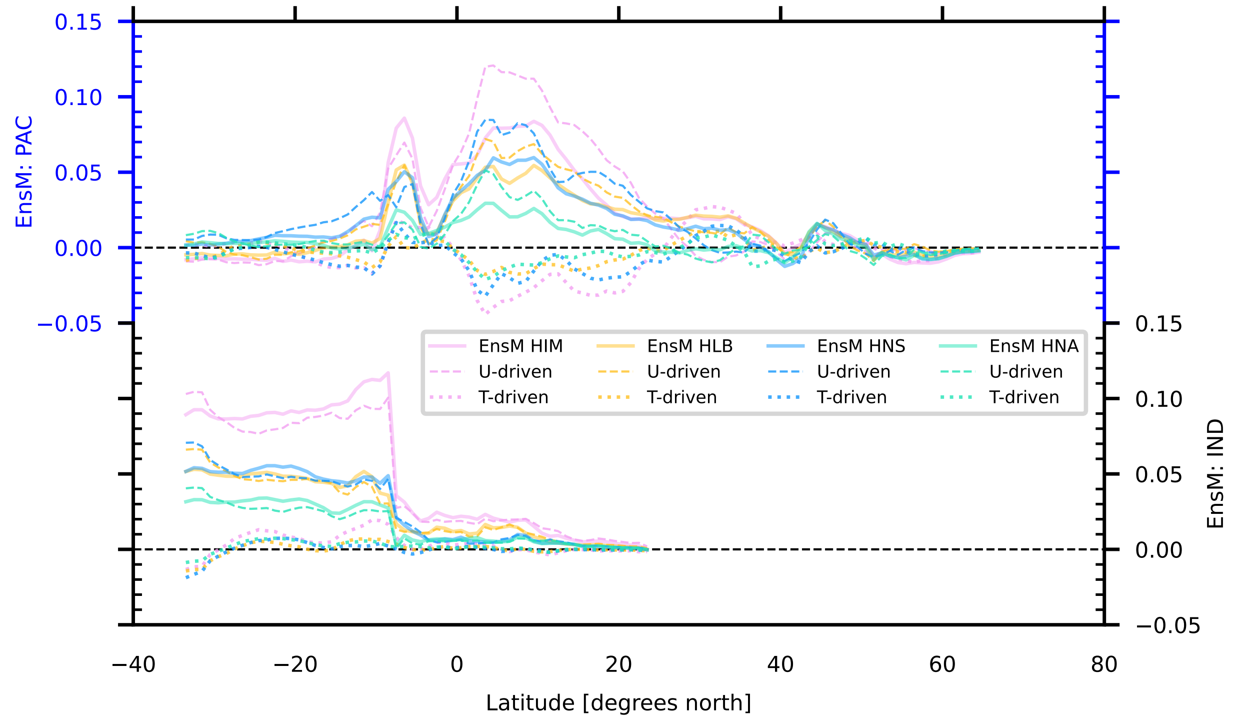

Fig. S4: **Changes in Meridional Heat Transport in the Indian Ocean (IND) and Pacific Ocean (PAC) Basins.** The solid lines represent the decomposition of meridional heat transport changes in the Indo-Pacific Basin, as shown in Fig. 3b of the main text, further broken down into the Indian and Pacific Oceans. The dashed and dotted lines represent the attribution of these changes to circulation-driven (U-driven) and temperature-driven (T-driven) components, respectively.

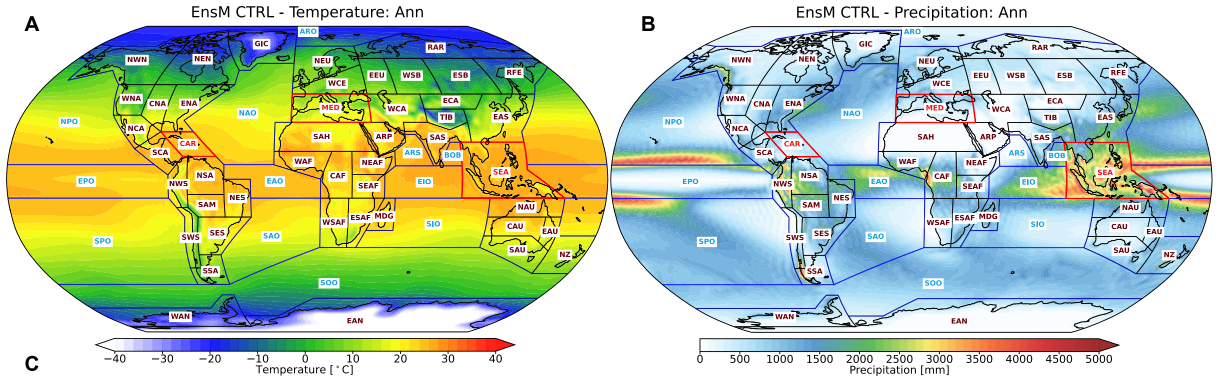

C

#	Abbr.	Region	#	Abbr.	Region	#	Abbr.	Region
0	GIC	Greenland/Iceland	20	SAH	Sahara	40	CAU	C.Australia
1	NWN	N.W.North-America	21	WAF	Western-Africa	41	EAU	E.Australia
2	NEN	N.E.North-America	22	CAF	Central-Africa	42	SAU	S.Australia
3	WNA	W.North-America	23	NEAF	N.Eastern-Africa	43	NZ	New-Zealand
4	CNA	C.North-America	24	SEAF	S.Eastern-Africa	44	EAN	E.Antarctica
5	ENA	E.North-America	25	WSAF	W.Southern-Africa	45	WAN	W.Antarctica
6	NCA	N.Central-America	26	ESAF	E.Southern-Africa			
7	SCA	S.Central-America	27	MDG	Madagascar	**46**	**ARO**	**Arctic-Ocean**
8	CAR	Caribbean	28	RAR	Russian-Arctic	**47**	**NPO**	**N.Pacific-Ocean**
9	NWS	N.W.South-America	29	WSB	W.Siberia	**48**	**EPO**	**Equatorial.Pacific-Ocean**
10	NSA	N.South-America	30	ESB	E.Siberia	**49**	**SPO**	**S.Pacific-Ocean**
11	NES	N.E.South-America	31	RFE	Russian-Far-East	**50**	**NAO**	**N.Atlantic-Ocean**
12	SAM	South-American-Monsoon	32	WCA	W.C.Asia	**51**	**EAO**	**Equatorial.Atlantic-Ocean**
13	SWS	S.W.South-America	33	ECA	E.C.Asia	**52**	**SAO**	**S.Atlantic-Ocean**
14	SES	S.E.South-America	34	TIB	Tibetan-Plateau	**53**	**ARS**	**Arabian-Sea**
15	SSA	S.South-America	35	EAS	E.Asia	**54**	**BOB**	**Bay-of-Bengal**
16	NEU	N.Europe	36	ARP	Arabian-Peninsula	**55**	**EIO**	**Equatorial.Indic-Ocean**
17	WCE	West&Central-Europe	37	SAS	S.Asia	**56**	**SIO**	**S.Indic-Ocean**
18	EEU	E.Europe	38	SEA	S.E.Asia	**57**	**SOO**	**Southern-Ocean**
19	MED	Mediterranean	39	NAU	N.Australia			

Note: *N. America (#1-5), C. America (#6-8), S. America (#9-15), Europe(#0,#16-19), Africa (#20-27), Asia (#28-38), Australasia (#39-43), Antarctica (#44-45), Ocean (#46-57)*

Fig. S5: **The climatological annual mean temperature and precipitation in control simulations.** Panels are shown as the ensemble mean of **(A)** temperature and **(B)** precipitation. **(C)** The IPCC AR6 reference regions across the globe. Annotated texts in **(A)** and **(B)** are the acronyms of each IPCC AR6 reference regions defined in **(C)**. In **(C)**, the regions indicated by red texts are considered both land and ocean regions, and the blue texts represent the defined ocean basins, so as the colored lines presented in **(A)** and **(B)** displaying the boundaries for the defined subcontinental regions.

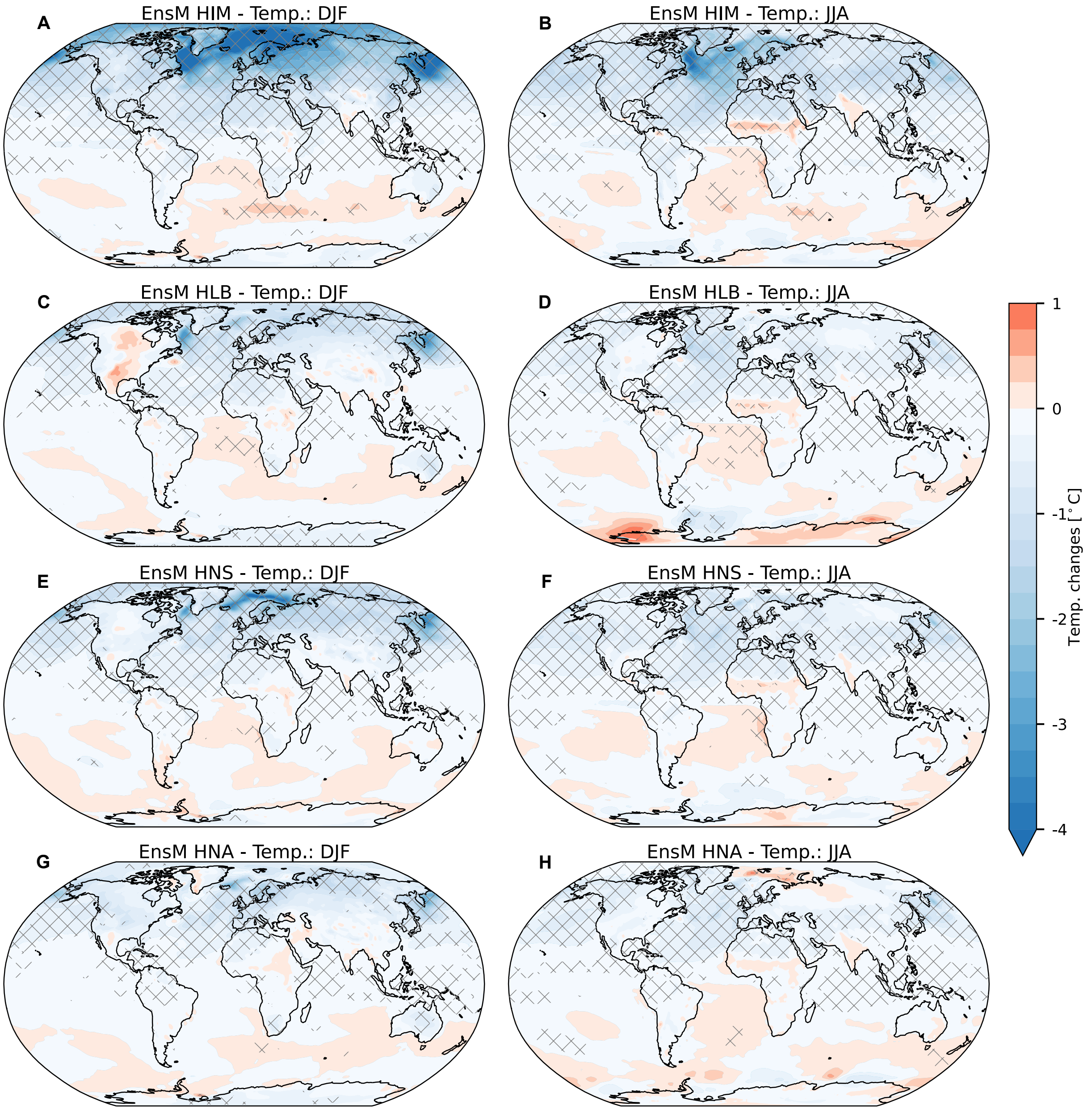

Fig. S6: **Response of surface air temperature during DJF and JJA in the four water-hosing experiments.** Shown as the difference between the perturbed and control experiments. **(A-B)** The anomalies for the HIM in winter (DJF) and summer (JJA), respectively. Same as **(A-B)**, **(C-D)** for HLB, **(E-F)** for HNS, and **(G-H)** for HNA. Hatched areas indicate significance at the 99% confidence level using a two-side Student's t-test.

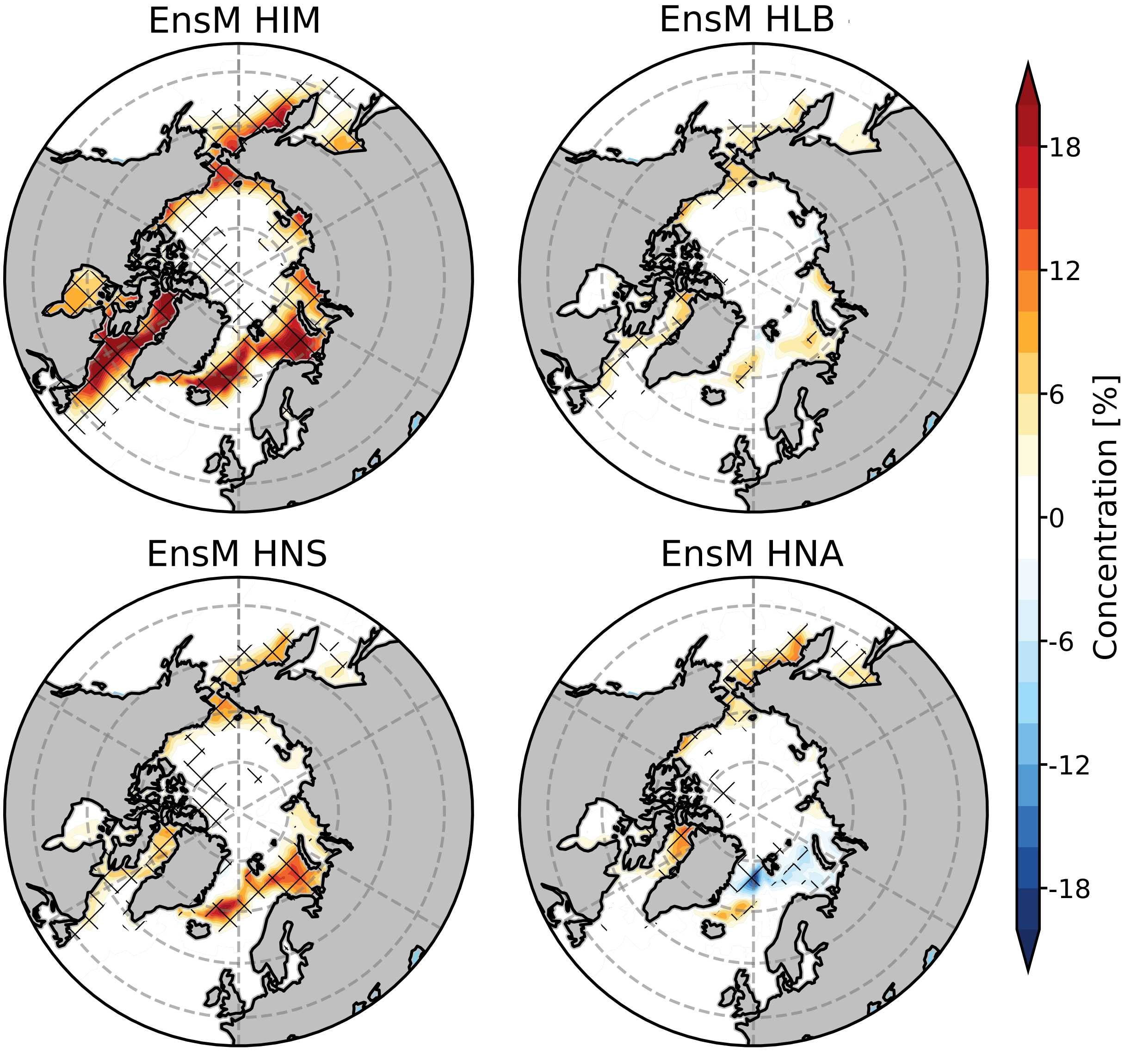

Fig. S7: **Changes in the sea-ice concentration during JJA.**

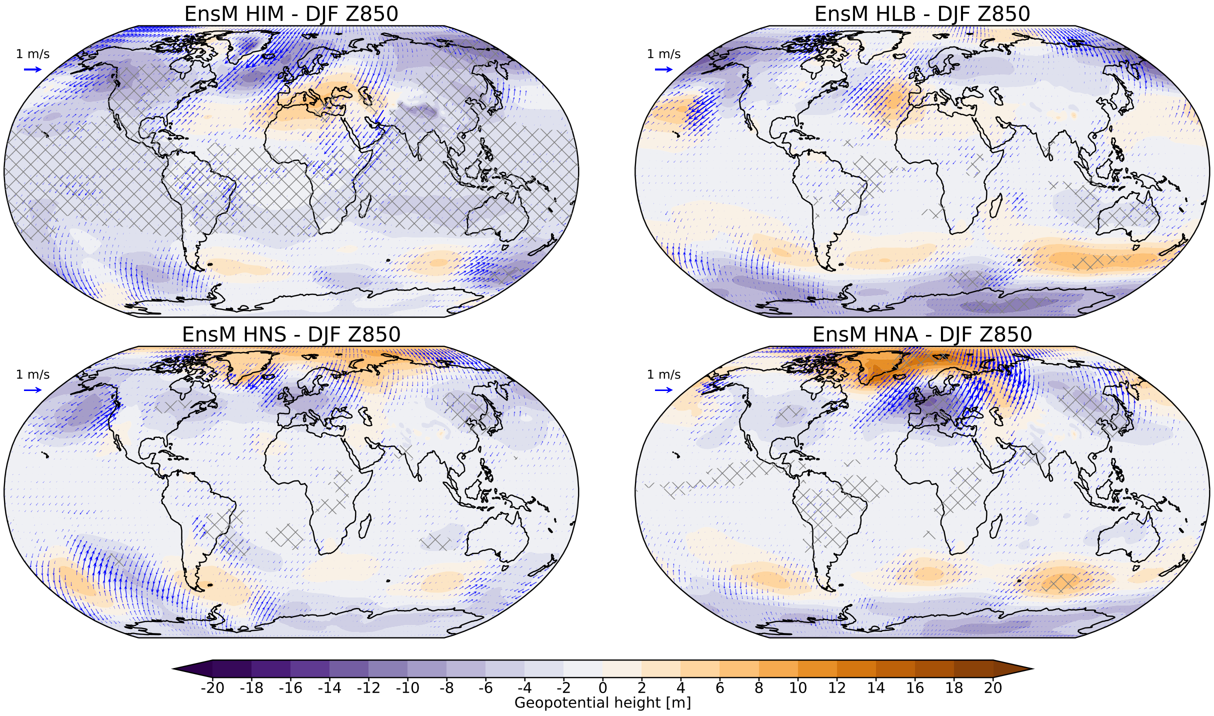

Fig. S8: **Changes in the geopotential height and winds at 850 hPa during DJF.**

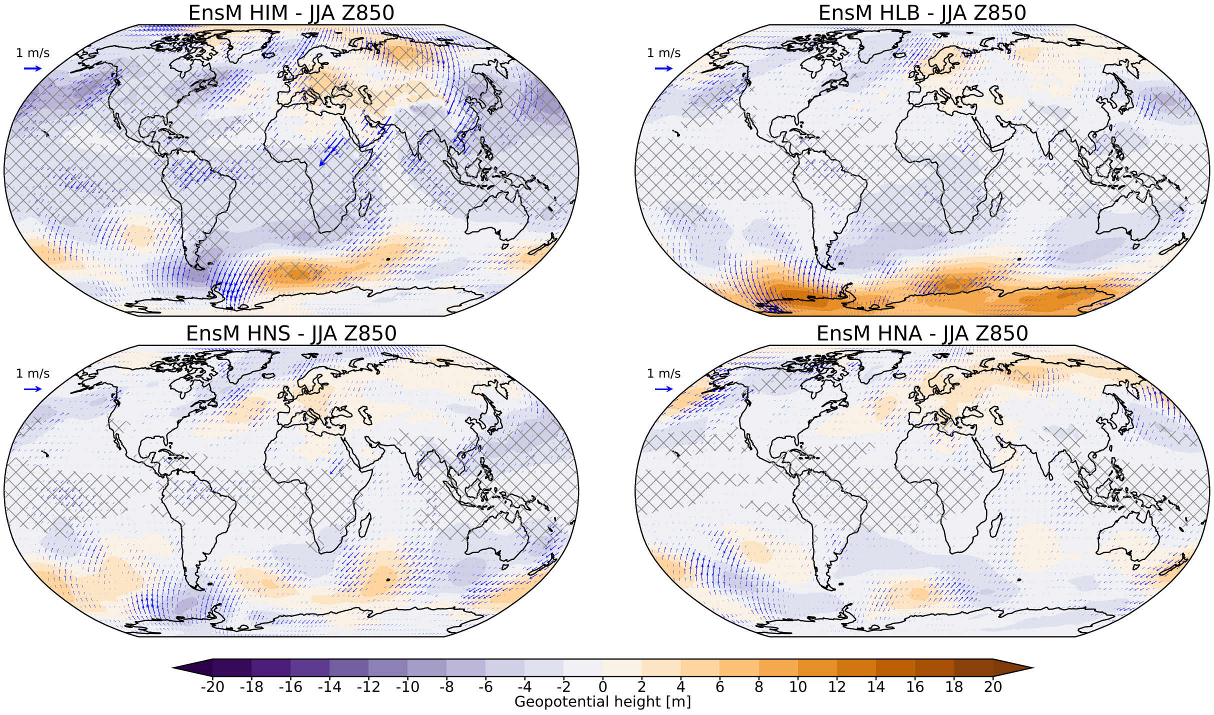

Fig. S9: **Changes in the geopotential height and winds at 850 hPa during JJA.**

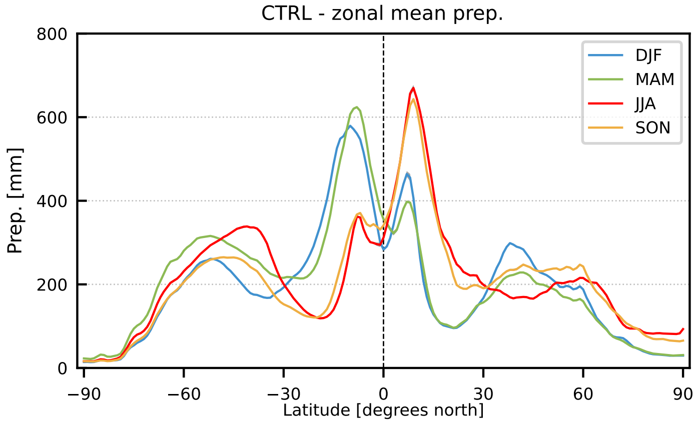

Fig. S10: **Annual mean zonal mean of seasonal precipitation in control simulation.** Shading indicates the ensemble spread measured by the ± 1 standard deviations.

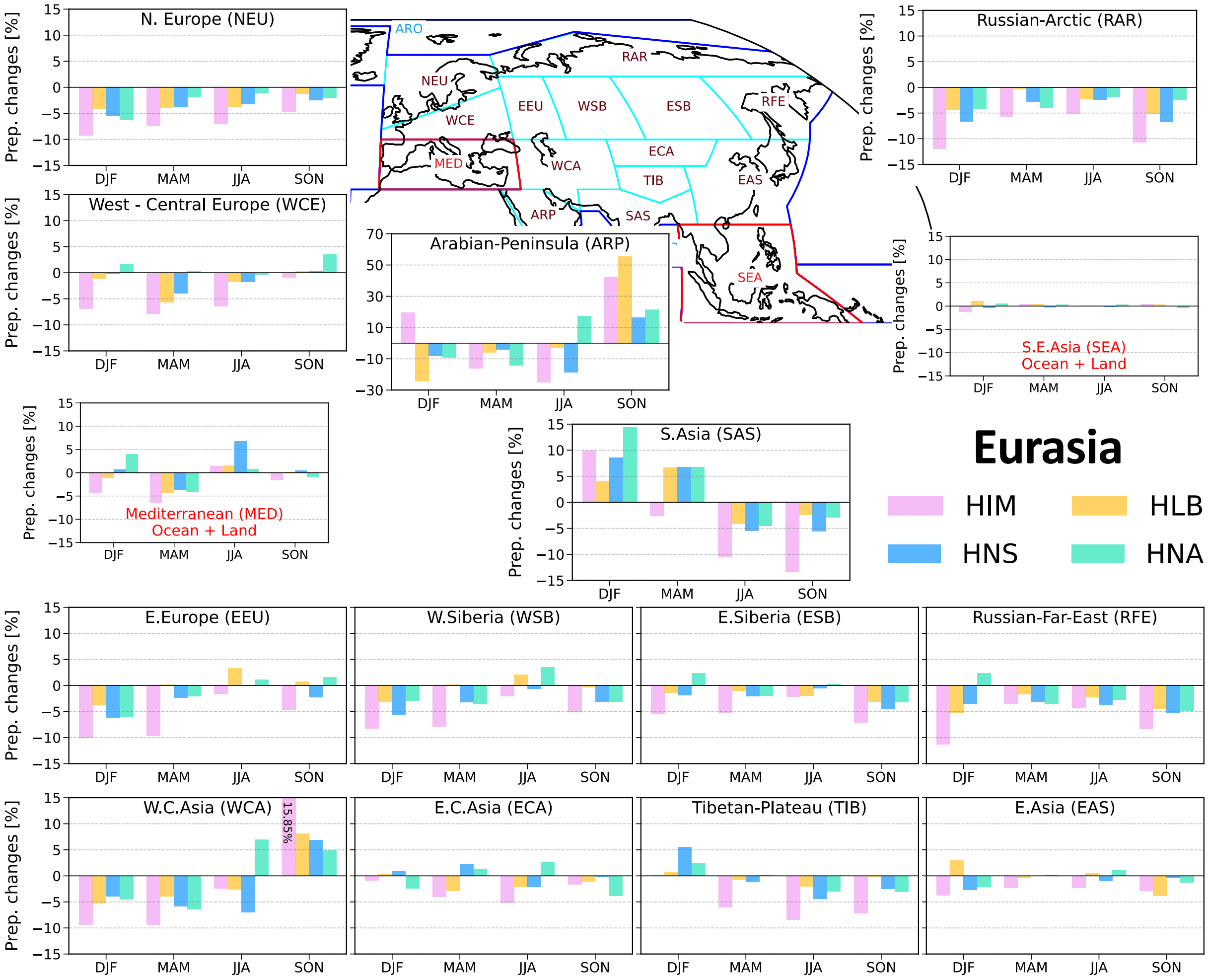

Fig. S11: **The relative changes of precipitation among different perturbed experiments over Eurasia.**

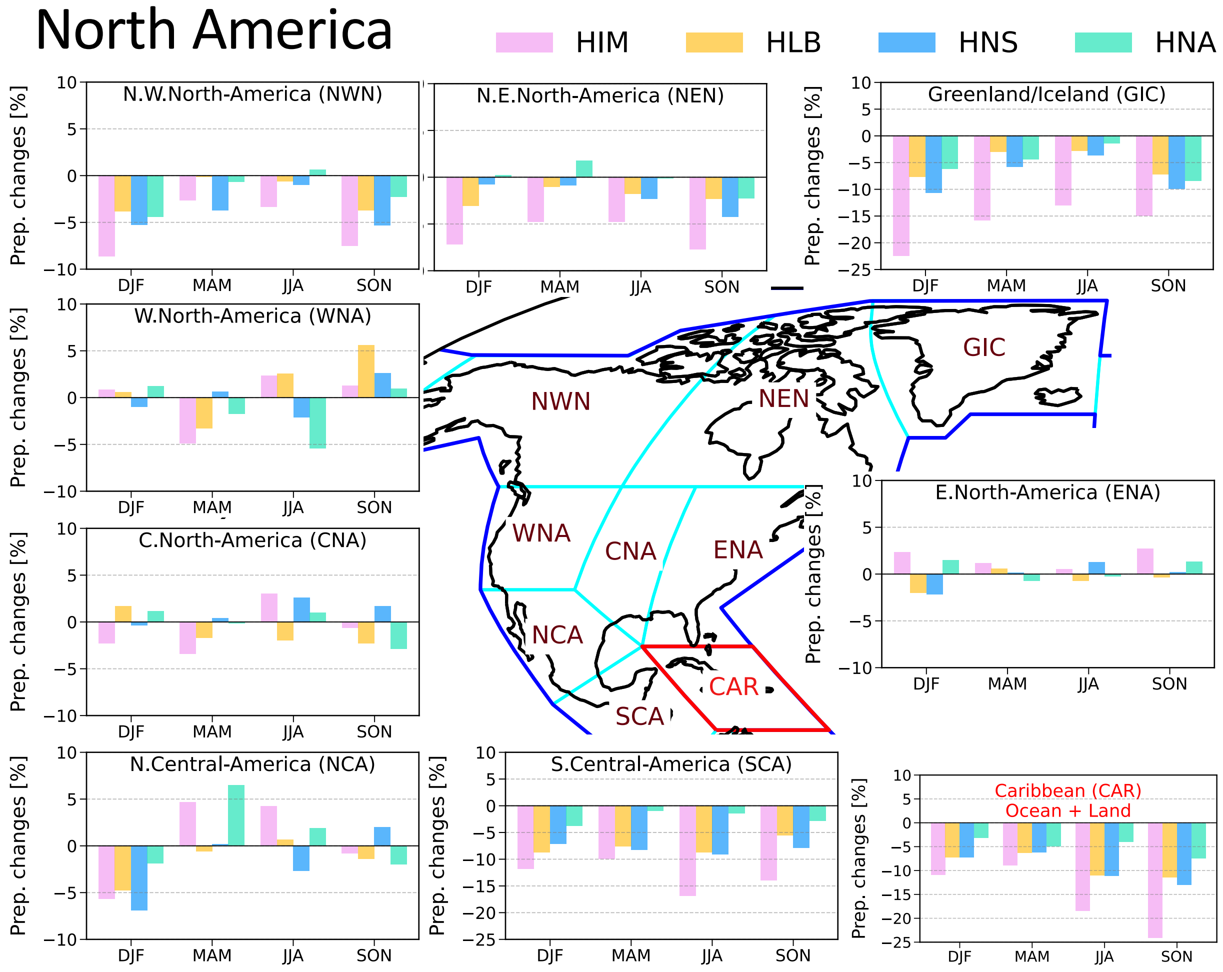

Fig. S12: **Same as Fig. S11, but for the North America and Greenland**

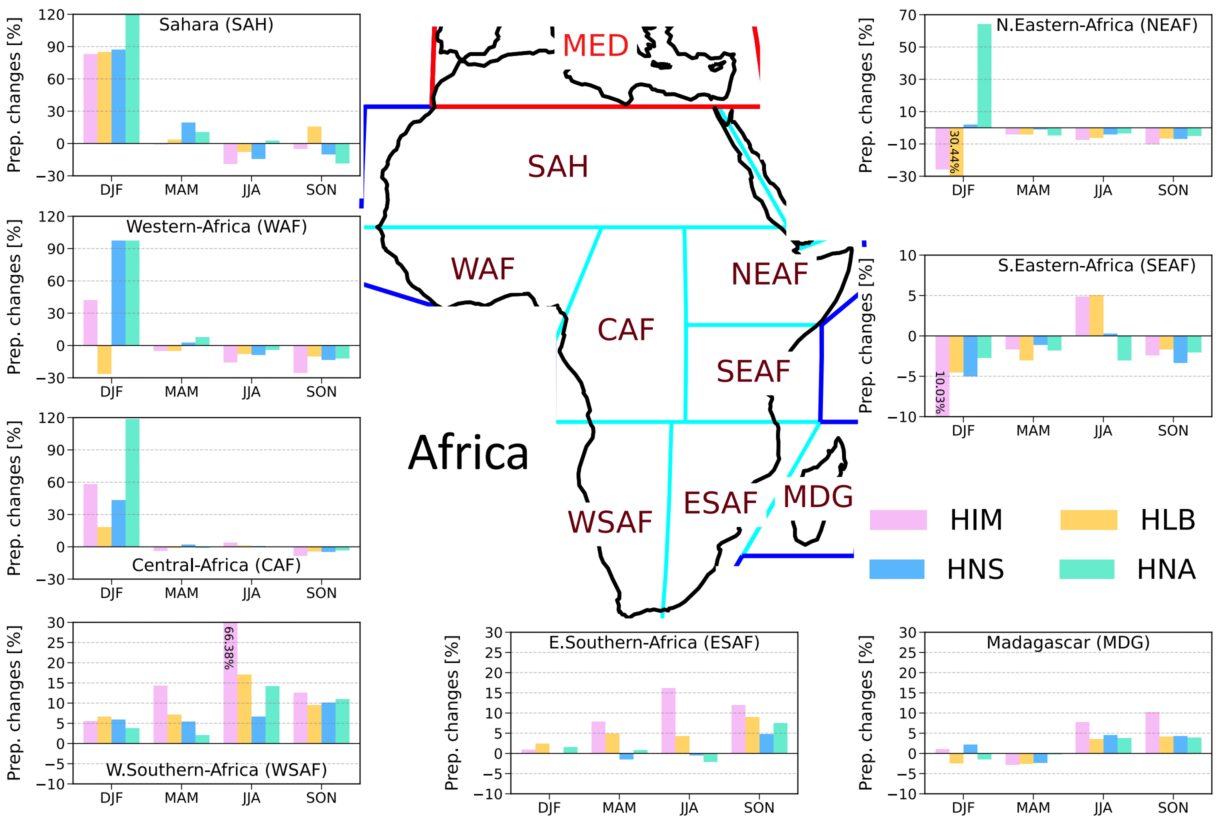

Fig. S13: **Same as Fig. S11, but for the Africa**

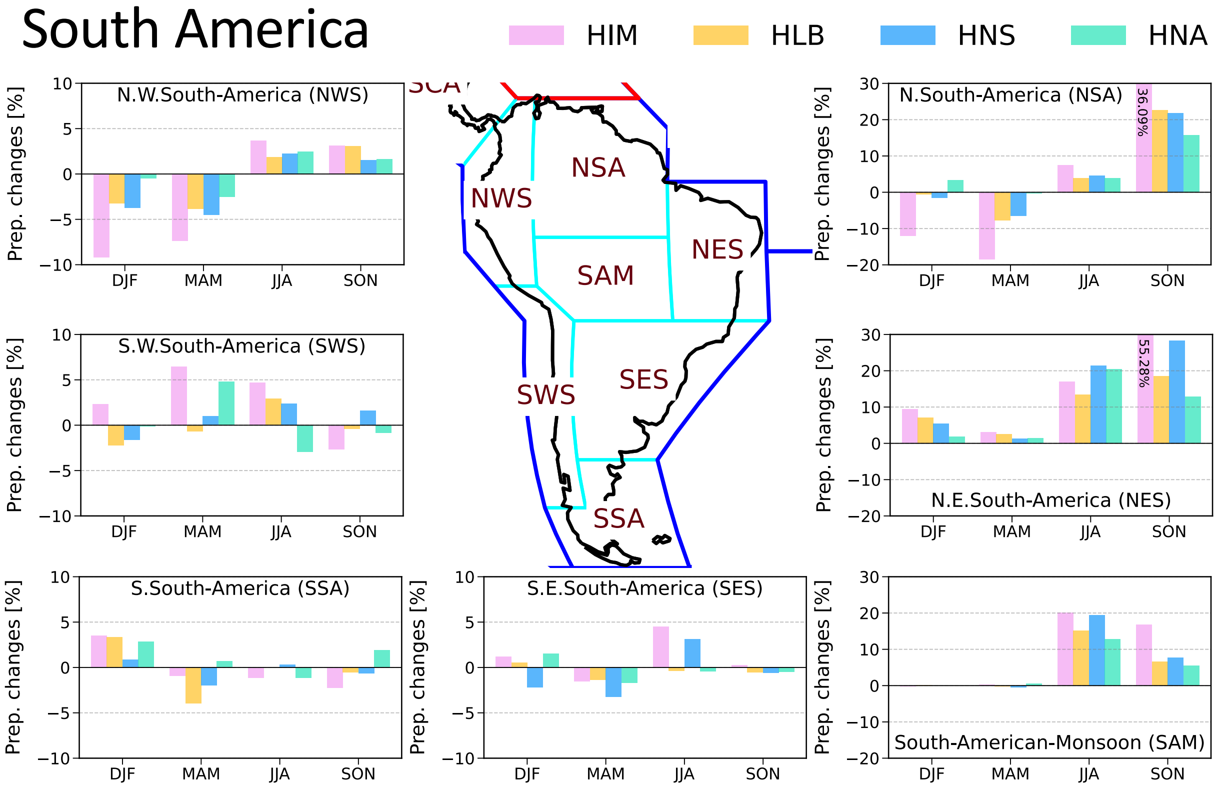

Fig. S14: **Same as Fig. S11, but for the South America**

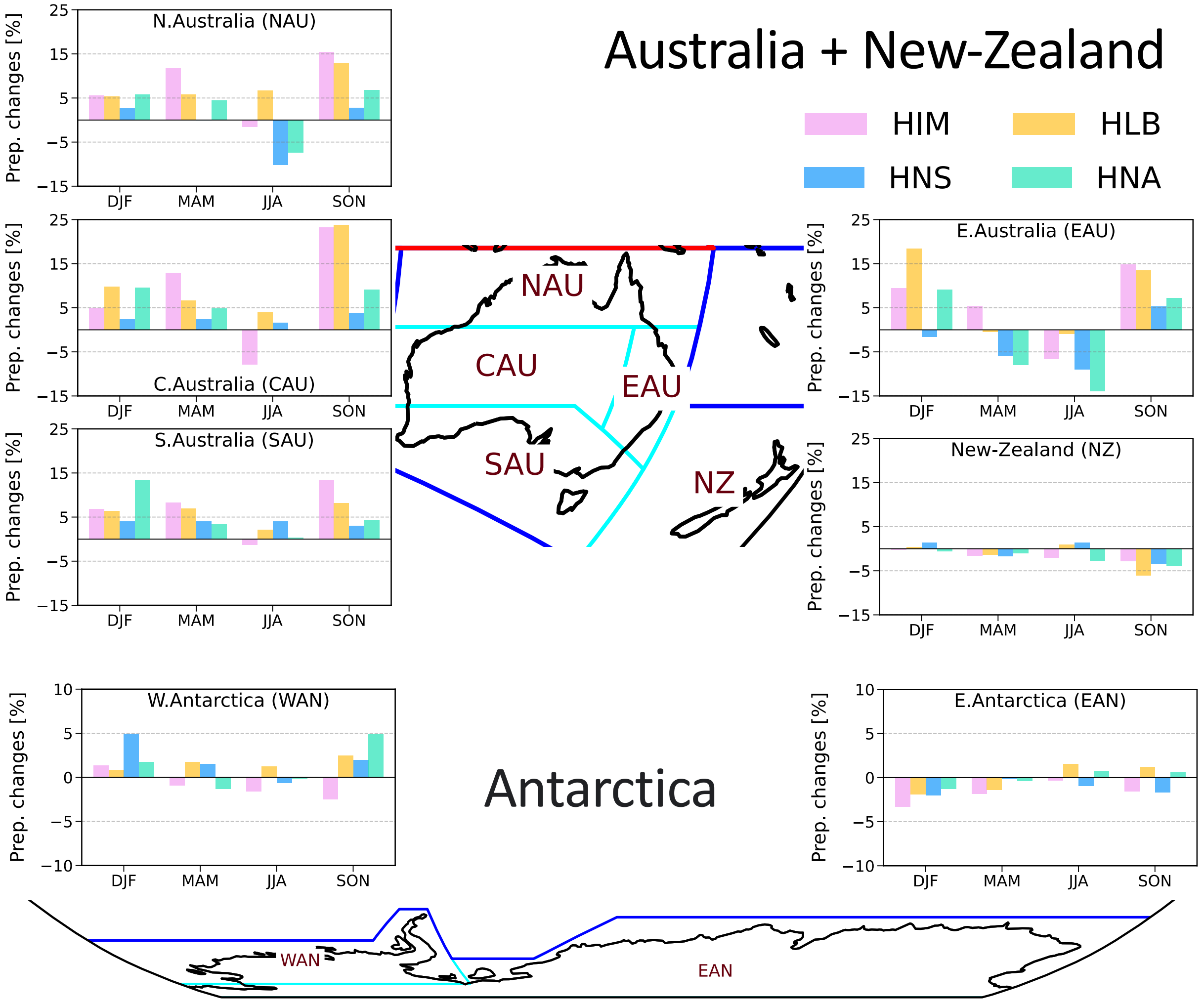

Fig. S15: **Same as Fig. S11, but for the Australia, New Zealand, and Antarctica.**

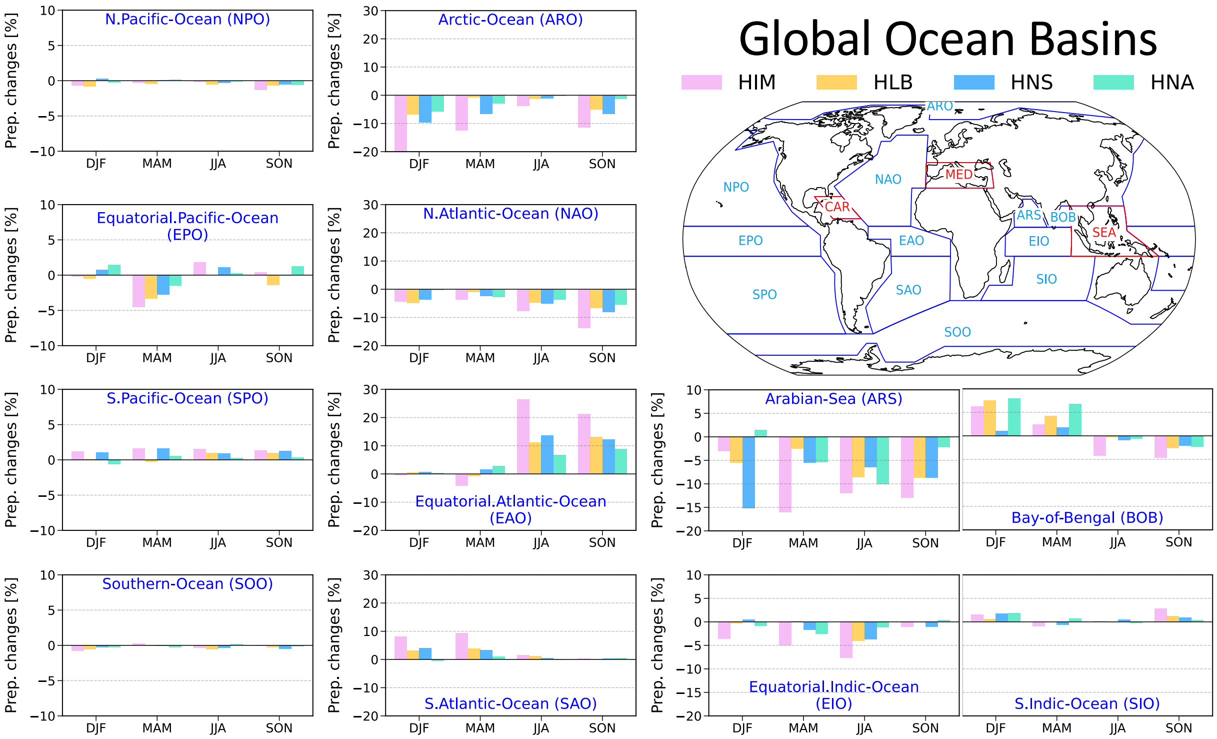

Fig. S16: **Same as Fig. S11, but for the global ocean basins.**

Table S1: Regions for the water-hosing experiments.

Basin	Abbreviation	Zonal ranges	Meridional ranges
Irminger Basin	HIM	52°N-70°N	40°W - 20°W
Labrador Sea	HLB	47°N-65°N	70°W - 40°W
Nordic Seas	HNS	65°N-85°N	20°W - 20°E
North-east Atlantic	HNA	47°N-65°N	20°W - 0°W

REFERENCES AND NOTES

1. L. C. Jackson, A. Biastoch, M. W. Buckley, D. G. Desbruyères, E. Frajka-Williams, B. Moat, and J. Robson, The evolution of the North Atlantic meridional overturning circulation since 1980. *Nat. Rev. Earth Environ.* **3**, 241–254 (2022).

2. B. Orihuela-Pinto, M. H. England, A. S. Taschetto, Interbasin and interhemispheric impacts of a collapsed Atlantic Overturning Circulation. *Nat. Clim. Change* **12**, 558–565 (2022).

3. W. Liu, A. V. Fedorov, S.-P. Xie, S. Hu, Climate impacts of a weakened Atlantic Meridional Overturning Circulation in a warming climate. *Sci. Adv.* **6**, eaaz4876 (2020).

4. E. Frajka-Williams, N. Foukal, G. Danabasoglu, Should AMOC observations continue: How and why?. *Philos. Trans. A Math. Phys. Eng. Sci.* **381**, 20220195 (2023).

5. F. Liu, X. Li, Y. Luo, W. Cai, J. Lu, X.-T. Zheng, S. M. Kang, H. Wang, L. Zhou, Increased Asian aerosols drive a slowdown of Atlantic Meridional Overturning Circulation. *Nat. Commun.* **15**, 18 (2024).

6. S. Rahmstorf, Ocean circulation and climate during the past 120,000 years. *Nature* **419**, 207–214 (2002).

7. A. C. Clement, L. C. Peterson, Mechanisms of abrupt climate change of the last glacial period. *Rev. Geophys.* **46**, RG4002 (2008).

8. W. Dansgaard, S. J. Johnsen, H. B. Clausen, D. Dahl-Jensen, N. S. Gundestrup, C. U. Hammer, C. S. Hvidberg, J. P. Steffensen, A. Sveinbjörnsdottir, J. Jouzel, G. Bond, Evidence for general instability of past climate from a 250-kyr ice-core record. *Nature* **364**, 218–220 (1993).

9. W. S. Broecker, Does the trigger for abrupt climate change reside in the ocean or in the atmosphere? *Science* **300**, 1519–1522 (2003).

10. P. U. Clark, N. G. Pisias, T. F. Stocker, A. J. Weaver, The role of the thermohaline circulation in abrupt climate change. *Nature* **415**, 863–869 (2002).

11. S. Rahmstorf, J. E. Box, G. Feulner, M. E. Mann, A. Robinson, S. Rutherford, E. J. Schaffernicht, Exceptional twentieth-century slowdown in Atlantic Ocean overturning circulation. *Nat. Clim. Change* **5**, 475–480 (2015).

12. L. Caesar, S. Rahmstorf, A. Robinson, G. Feulner, V. Saba, Observed fingerprint of a weakening Atlantic Ocean overturning circulation. *Nature* **556**, 191–196 (2018).

13. X. Chen, K.-K. Tung, Global surface warming enhanced by weak Atlantic overturning circulation. *Nature* **559**, 387–391 (2018).

14. D. J. Thornalley, D. W. Oppo, P. Ortega, J. I. Robson, C. M. Brierley, R. Davis, I. R. Hall, P. Moffa-Sanchez, N. L. Rose, P. T. Spooner, I. Yashayaev, L. D. Keigwin, Anomalously weak Labrador Sea convection and Atlantic overturning during the past 150 years. *Nature* **556**, 227–230 (2018).

15. T. Semmler, S. Danilov, P. Gierz, H. F. Goessling, J. Hegewald, C. Hinrichs, N. Koldunov, N. Khosravi, L. Mu, T. Rackow, D. V. Sein, D. Sidorenko, Q. Wang, T. Jung, Simulations for CMIP6 with the AWI climate model AWI-CM-1-1. *J. Adv. Model. Earth Syst.* **12**, e2019MS002009 (2020).

16. V. Masson-Delmotte, P. Zhai, A. Pirani, S. L. Connors, C. Péan, S. Berger, N. Caud, Y. Chen, L. Goldfarb, M. I. Gomis, *M. Huang*, K. Leitzell, E. Lonnoy, J. B. R. Matthews, T. K. Maycock, T. Waterfield, O. Yelekçi, R. Yu, B. Zhou, *Climate change 2021: The physical science basis* (IPCC, 2021).

17. W. Liu, S.-P. Xie, Z. Liu, J. Zhu, Overlooked possibility of a collapsed Atlantic Meridional Overturning Circulation in warming climate. *Sci. Adv.* **3**, e1601666 (2017).

18. L. C. Jackson, R. Kahana, T. Graham, M. Ringer, T. Woollings, J. Mecking, R. Wood, Global and European climate impacts of a slowdown of the AMOC in a high resolution GCM. *Clim. Dyn.* **45**, 3299–3316 (2015).

19. X. Shi, G. Lohmann, D. Sidorenko, H. Yang, Early-Holocene simulations using different forcings and resolutions in AWI-ESM. *Holocene* **30**, 996–1015 (2020).

20. G. Lohmann, Atmospheric and oceanic freshwater transport during weak Atlantic overturning circulation. *Tellus A* **55**, 438–449 (2003).

21. T. Kleinen, T. J. Osborn, K. R. Briffa, Sensitivity of climate response to variations in freshwater hosing location. *Ocean Dyn.* **59**, 509–521 (2009).

22. L. Yu, Y. Gao, O. H. Otterå, The sensitivity of the Atlantic meridional overturning circulation to enhanced freshwater discharge along the entire, eastern and western coast of Greenland. *Clim. Dyn.* **46**, 1351–1369 (2016).

23. L. C. Jackson, E. Alastrué de Asenjo, K. Bellomo, G. Danabasoglu, H. Haak, A. Hu, J. Jungclaus, W. Lee, V. L. Meccia, O. Saenko, A. Shao, D. Swingedouw, Understanding AMOC stability: The North Atlantic hosing model intercomparison project. *Geosci. Model Dev.* **16**, 1975–1995 (2023).

24. R. S. Smith, J. M. Gregory, A study of the sensitivity of ocean overturning circulation and climate to freshwater input in different regions of the North Atlantic. *Geophys. Res. Lett.* **36**, L15701 (2009).

25. G. Lohmann, M. Butzin, N. Eissner, X. Shi, C. Stepanek, Abrupt climate and weather changes across time scales. *Paleoceanogr. Paleoclimatol.* **35**, e2019PA003782 (2020).

26. F. Sévellec, A. V. Fedorov, W. Liu, Arctic sea-ice decline weakens the Atlantic meridional overturning circulation. *Nat. Clim. Change* **7**, 604–610 (2017).

27. C. W. Böning, E. Behrens, A. Biastoch, K. Getzlaff, J. L. Bamber, Emerging impact of Greenland meltwater on deepwater formation in the North Atlantic Ocean. *Nat. Geosci.* **9**, 523–527 (2016).

28. W. Liu, A. Fedorov, Interaction between Arctic sea ice and the Atlantic meridional overturning circulation in a warming climate. *Clim. Dyn.* **58**, 1811–1827 (2022).

29. W. Liu, A. Fedorov, F. Sévellec, The mechanisms of the Atlantic meridional overturning circulation slowdown induced by Arctic sea ice decline. *J. Climate* **32**, 977–996 (2019).

30. D. M. Chandler, A. Hubbard, Widespread partial-depth hydrofractures in ice sheets driven by supraglacial streams. *Nat. Geosci.* **16**, 605–611 (2023).

31. F. He, P. U. Clark, Freshwater forcing of the atlantic meridional overturning circulation revisited. *Nat. Clim. Change* **12**, 449–454 (2022).

32. C. Lique, M. D. Thomas, Latitudinal shift of the Atlantic Meridional Overturning Circulation source regions under a warming climate. *Nat. Clim. Change* **8**, 1013–1020 (2018).

33. L. Chafik, T. Rossby, Volume, heat, and freshwater divergences in the subpolar North Atlantic suggest the Nordic Seas as key to the state of the meridional overturning circulation. *Geophys. Res. Lett.* **46**, 4799–4808 (2019).

34. A. Megann, A. Blaker, S. Josey, A. New, B. Sinha, Mechanisms for late 20th and early 21st century decadal AMOC variability. *J. Geophys. Res. Oceans* **126**, e2021JC017865 (2021).

35. J. Streffing, D. Sidorenko, T. Semmler, L. Zampieri, P. Scholz, M. Andrés-Martínez, N. Koldunov, T. Rackow, J. Kjellsson, H. Goessling, M. Athanase, Q. Wang, J. Hegewald, D. V. Sein, L. Mu, U. Fladrich, D. Barbi, P. Gierz, S. Danilov, S. Juricke, G. Lohmann, T. Jung, AWI-CM3 coupled climate model: description and evaluation experiments for a prototype post-CMIP6 model. *Geosci. Model Dev.* **15**, 6399–6427 (2022).

36. L. Chafik, N. P. Holliday, S. Bacon, T. Rossby, Irminger Sea is the center of action for subpolar AMOC variability. *Geophys. Res. Lett.* **49**, e2022GL099133 (2022).

37. M. B. Menary, T. Kuhlbrodt, J. Ridley, M. B. Andrews, O. B. Dimdore-Miles, J. Deshayes, R. Eade, L. Gray, S. Ineson, J. Mignot, C. D. Roberts, J. Robson, R. A. Wood, P. Xavier, Preindustrial control simulations with HadGEM3-GC3.1 for CMIP6. *J. Adv. Model. Earth Syst.* **10**, 3049–3075 (2018).

38. E. Rousi, F. Selten, S. Rahmstorf, D. Coumou, Changes in North Atlantic atmospheric circulation in a warmer climate favor winter flooding and summer drought over Europe. *J. Climate* **34**, 2277–2295 (2021).

39. E. Frajka-Williams, I. J. Ansorge, J. Baehr, H. L. Bryden, M. P. Chidichimo, S. A. Cunningham, G. Danabasoglu, S. Dong, K. A. Donohue, S. Elipot, P. Heimbach, N. P. Holliday, R. Hummels, L. C. Jackson, J. Karstensen, M. Lankhorst, I. A. Le Bras, M. S. Lozier, E. L. McDonagh, C. S. Meinen, H. Mercier, B. I. Moat, R. C. Perez, C. G. Piecuch, M. Rhein, M. A. Srokosz, K. E. Trenberth, S. Bacon, G. Forget, G. Goni, D. Kieke, J. Koelling, T. Lamont, G. D. McCarthy, C. Mertens, U. Send, D. A. Smeed, S. Speich, M. van den Berg, D. Volkov, C. Wilson, Atlantic meridional overturning circulation: Observed transport and variability. *Front. Mar. Sci.* **6**, 260 (2019).

40. G. I. Monterey, S. Levitus, Seasonal variability of mixed layer depth for the world ocean (NOAA, 1997), 100 pp.

41. A. M. Treguier, C. de Boyer Montégut, A. Bozec, E. P. Chassignet, B. Fox-Kemper, A. McC Hogg, D. Iovino, A. E. Kiss, J. Le Sommer, Y. Li, P. Lin, C. Lique, H. Liu, G. Serazin, D. Sidorenko, Q. Wang, X. Xu, S. Yeager, The mixed-layer depth in the Ocean Model Intercomparison Project (OMIP): Impact of resolving mesoscale eddies. *Geosci. Model Dev.* **16**, 3849–3872 (2023).

42. K. E. Trenberth, J. M. Caron, Estimates of meridional atmosphere and ocean heat transports. *J. Climate* **14**, 3433–3443 (2001).

43. H. Yang, Q. Li, K. Wang, Y. Sun, D. Sun, Decomposing the meridional heat transport in the climate system. *Clim. Dyn.* **44**, 2751–2768 (2015).

44. W. E. Johns, M. O. Baringer, L. M. Beal, S. A. Cunningham, T. Kanzow, H. L. Bryden, J. J. M. Hirschi, J. Marotzke, C. S. Meinen, B. Shaw, R. Curry, Continuous, array-based estimates of Atlantic Ocean heat transport at 26.5°N. *J. Climate* **24**, 2429–2449 (2011).

45. R. J. Stouffer, J. Yin, J. M. Gregory, K. W. Dixon, M. J. Spelman, W. Hurlin, A. J. Weaver, M. Eby, G. M. Flato, H. Hasumi, A. Hu, J. H. Jungclaus, I. V. Kamenkovich, A. Levermann, M. Montoya, S. Murakami, S. Nawrath, A. Oka, W. R. Peltier, D. Y. Robitaille, A. Sokolov, G. Vettoretti, S. L. Weber, Investigating the causes of the response of the thermohaline circulation to past and future climate changes. *J. Climate* **19**, 1365–1387 (2006).

46. R. Zhang, Latitudinal dependence of Atlantic meridional overturning circulation (AMOC) variations. *Geophys. Res. Lett.* **37**, L16703 (2010).

47. D. Sidorenko, S. Danilov, J. Streffing, V. Fofonova, H. F. Goessling, P. Scholz, Q. Wang, A. Androsov, W. Cabos, S. Juricke, N. Koldunov, T. Rackow, D. V. Sein, T. Jung, AMOC variability and watermass transformations in the AWI climate model. *J. Adv. Model. Earth Syst.* **13**, e2021MS002582 (2021).

48. C. Zhu, L. Cheng, Sensitivity of AMOC fingerprints under future anthropogenic warming. *Geophys. Res. Lett.* **51**, e2023GL107170 (2024).

49. M. S. Lozier, F. Li, S. Bacon, F. Bahr, A. S. Bower, S. A. Cunningham, M. F. de Jong, L. de Steur, B. deYoung, J. Fischer, S. F. Gary, B. J. W. Greenan, N. P. Holliday, A. Houk, L. Houpert, M. E. Inall, W. E. Johns, H. L. Johnson, C. Johnson, J. Karstensen, G. Koman, I. A. Le Bras, X. Lin, N. Mackay, D. P. Marshall, H. Mercier, M. Oltmanns, R. S. Pickart, A. L. Ramsey, D. Rayner, F. Straneo, V. Thierry, D. J. Torres, R. G. Williams, C. Wilson, J. Yang, I. Yashayaev, J. Zhao, A sea change in our view of overturning in the subpolar North Atlantic. *Science* **363**, 516–521 (2019).

50. D. Sidorenko, S. Danilov, V. Fofonova, W. Cabos, N. Koldunov, P. Scholz, D. V. Sein, Q. Wang, AMOC, water mass transformations, and their responses to changing resolution in the Finite-volumE Sea ice-Ocean model. *J. Adv. Model. Earth Syst.* **12**, e2020MS002317 (2020).

51. S. Li, W. Liu, Deciphering the migration of the intertropical convergence zone during the last deglaciation. *Geophys. Res. Lett.* **49**, e2022GL098806 (2022).

52. E. R. Newsom, A. F. Thompson, Reassessing the role of the Indo-Pacific in the ocean’s global overturning circulation. *Geophys. Res. Lett.* **45**, 12,422–12,431 (2018).

53. S. Sun, A. F. Thompson, S.-P. Xie, S.-M. Long, Indo-Pacific warming induced by a weakening of the Atlantic meridional overturning circulation. *J. Climate* **35**, 815–832 (2022).

54. S. Hu, A. V. Fedorov, Indian Ocean warming can strengthen the Atlantic meridional overturning circulation. *Nat. Clim. Chang.* **9**, 747–751 (2019).

55. S. Li, W. Liu, Impacts of Arctic sea ice loss on global ocean circulations and interbasin ocean heat exchanges. *Clim. Dyn.* **59**, 2701–2716 (2022).

56. R. Zhang, M. Thomas, Horizontal circulation across density surfaces contributes substantially to the long-term mean northern Atlantic Meridional Overturning Circulation. *Commun. Earth Environ.* **2**, 112 (2021).

57. M. Vellinga, R. A. Wood, Global climatic impacts of a collapse of the Atlantic thermohaline circulation. *Clim. Chang.* **54**, 251–267 (2002).

58. M. Iturbide, J. M. Gutiérrez, L. M. Alves, J. Bedia, E. Cimadevilla, A. S. Cofiño, R. Cerezo-Mota, A. Di Luca, S. H. Faria, I. Gorodetskaya, M. Hauser, S. Herrera, K. Hennessy, H. T. Hewitt, R. G. Jones, S. Krakovska, R. Manzanas, D. Martínez-Castro, G. T. Narisma, I. S. Nurhati, I. Pinto, S. I. Seneviratne, B. van den Hurk, C. S. Vera, An update of IPCC climate reference regions for subcontinental analysis of climate model data: definition and aggregated datasets. *Earth Syst. Sci. Data* **12**, 2959–2970 (2020).

59. P. Chang, R. Zhang, W. Hazeleger, C. Wen, X. Wan, L. Ji, R. J. Haarsma, W.-P. Breugem, H. Seidel, Oceanic link between abrupt changes in the North Atlantic Ocean and the African monsoon. *Nat. Geosci.* **1**, 444–448 (2008).

60. S.-P. Xie, A dynamic ocean–atmosphere model of the tropical Atlantic decadal variability. *J. Climate* **12**, 64–70 (1999).

61. K. Bellomo, M. Angeloni, S. Corti, J. von Hardenberg, Future climate change shaped by inter-model differences in Atlantic meridional overturning circulation response. *Nat. Commun.* **12**, 3659 (2021).

62. D. Nian, S. Bathiany, M. Ben-Yami, L. L. Blaschke, M. Hirota, R. R. Rodrigues, N. Boers, A potential collapse of the Atlantic Meridional Overturning Circulation may stabilise eastern Amazonian rainforests. *Commun. Earth Environ.* **4**, 470 (2023).

63. D. McGee, E. Moreno-Chamarro, B. Green, J. Marshall, E. Galbraith, L. Bradtmiller, Hemispherically asymmetric trade wind changes as signatures of past ITCZ shifts. *Quat. Sci. Rev.* **180**, 214–228 (2018).

64. J. Yin, M. Zhao, Influence of the Atlantic meridional overturning circulation on the US extreme cold weather. *Commun. Earth Environ.* **2**, 218 (2021).

65. V. L. Meccia, C. Simolo, K. Bellomo, S. Corti, Extreme cold events in Europe under a reduced AMOC. *Environ. Res. Lett.* **19**, 014054 (2023).

66. M. Ionita, V. Nagavciuc, P. Scholz, M. Dima, Long-term drought intensification over Europe driven by the weakening trend of the Atlantic Meridional Overturning Circulation. *J. Hydrol. Reg. Stud.* **42**, 101176 (2022).

67. R. P. Allan, Amplified seasonal range in precipitation minus evaporation. *Environ. Res. Lett.* **18**, 094004 (2023).

68. P. Ditlevsen, S. Ditlevsen, Warning of a forthcoming collapse of the Atlantic meridional overturning circulation. *Nat. Commun.* **14**, 4254 (2023).

69. W. Weijer, W. Cheng, O. A. Garuba, A. Hu, B. Nadiga, CMIP6 models predict significant 21st century decline of the Atlantic meridional overturning circulation. *Geophys. Res. Lett.* **47**, e2019GL086075 (2020).

70. R. Sutton, G. D. McCarthy, J. Robson, B. Sinha, A. Archibald, L. Gray, Atlantic multidecadal variability and the UK ACSIS program. *Bull. Am. Meteorol. Soc.* **99**, 415–425 (2018).

71. K. Bellomo, V. L. Meccia, R. D'Agostino, F. Fabiano, S. M. Larson, J. von Hardenberg, S. Corti, Impacts of a weakened AMOC on precipitation over the Euro-Atlantic region in the EC-Earth3 climate model. *Clim. Dyn.* **61**, 3397–3416 (2023).

72. R. Zhang, R. Sutton, G. Danabasoglu, Y.-O. Kwon, R. Marsh, S. G. Yeager, D. E. Amrhein, C. M. Little, A review of the role of the Atlantic meridional overturning circulation in Atlantic multidecadal variability and associated climate impacts. *Rev. Geophys.* **57**, 316–375 (2019).

73. T. Woollings, J. M. Gregory, J. G. Pinto, M. Reyers, D. J. Brayshaw, Response of the North Atlantic storm track to climate change shaped by ocean–atmosphere coupling. *Nat. Geosci.* **5**, 313–317 (2012).

74. N. P. Holliday, M. Bersch, B. Berx, L. Chafik, S. Cunningham, C. Florindo-López, H. Hátún, W. Johns, S. A. Josey, K. M. H. Larsen, S. Mulet, M. Oltmanns, G. Reverdin, T. Rossby, V. Thierry, H. Valdimarsson, I. Yashayaev, Ocean circulation causes the largest freshening event for 120 years in eastern subpolar North Atlantic. *Nat. Commun.* **11**, 585 (2020).

75. I. Le Bras, F. Straneo, M. Muilwijk, L. H. Smedsrud, F. Li, M. S. Lozier, N. P. Holliday, How much Arctic fresh water participates in the subpolar overturning circulation? *J. Phys. Oceanogr.* **51**, 955–973 (2021).

76. S. Drijfhout, G. J. Van Oldenborgh, A. Cimatoribus, Is a decline of AMOC causing the warming hole above the North Atlantic in observed and modeled warming patterns? *J. Climate* **25**, 8373–8379 (2012).

77. M. B. Menary, R. A. Wood, An anatomy of the projected North Atlantic warming hole in CMIP5 models. *Clim. Dyn.* **50**, 3063–3080 (2018).

78. Q. Wang, S. Danilov, D. Sidorenko, R. Timmermann, C. Wekerle, X. Wang, T. Jung, J. Schröter, The Finite Element Sea Ice-Ocean Model (FESOM) v.1.4: Formulation of an ocean general circulation model. *Geosci. Model Dev.* **7**, 663–693 (2014).

79. P. Scholz, D. Sidorenko, O. Gurses, S. Danilov, N. Koldunov, Q. Wang, D. Sein, M. Smolentseva, N. Rakowsky, T. Jung, Assessment of the Finite-volumE Sea ice-Ocean Model (FESOM2.0)–Part 1: Description of selected key model elements and comparison to its predecessor version. *Geosci. Model Dev.* **12**, 4875–4899 (2019).

80. D. Sidorenko, H. F. Goessling, N. Koldunov, P. Scholz, S. Danilov, D. Barbi, W. Cabos, O. Gurses, S. Harig, C. Hinrichs, S. Juricke, G. Lohmann, M. Losch, L. Mu, T. Rackow, N. Rakowsky, D. Sein, T. Semmler, X. Shi, C. Stepanek, J. Streffing, Q. Wang, C. Wekerle, H.

Yang, T. Jung, Evaluation of FESOM2.0 coupled to ECHAM6.3: Preindustrial and HighResMIP simulations. *J. Adv. Model. Earth Syst.* **11**, 3794–3815 (2019).

81. R. K. Haskins, K. I. Oliver, L. C. Jackson, R. A. Wood, S. S. Drijfhout, Temperature domination of AMOC weakening due to freshwater hosing in two GCMs. *Clim. Dyn.* **54**, 273–286 (2020).

82. L. Jackson, R. Wood, Hysteresis and resilience of the AMOC in an eddy-permitting GCM. *Geophys. Res. Lett.* **45**, 8547–8556 (2018).

83. A. E. Gill, Some simple solutions for heat-induced tropical circulation. *Q. J. R. Meteorol. Soc.* **106**, 447–462 (1980).

84. X. Zhang, L. Alexander, G. C. Hegerl, P. Jones, A. K. Tank, T. C. Peterson, B. Trewin, F. W. Zwiers, Indices for monitoring changes in extremes based on daily temperature and precipitation data. *Wiley Interdiscip. Rev. Clim. Change* **2**, 851–870 (2011).

85. P. Wu, L. Jackson, A. Pardaens, N. Schaller, Extended warming of the northern high latitudes due to an overshoot of the Atlantic meridional overturning circulation. *Geophys. Res. Lett.* **38**, L24704 (2011).

86. R. K. Haskins, K. I. Oliver, L. C. Jackson, S. S. Drijfhout, R. A. Wood, Explaining asymmetry between weakening and recovery of the AMOC in a coupled climate model. *Clim. Dyn.* **53**, 67–79 (2019).
